# Variants Associated with the Ankle Brachial Index Differ by Hispanic/Latino Ethnic Group: a genome-wide association study in the Hispanic Community Health Study/Study of Latinos

**DOI:** 10.1038/s41598-019-47928-5

**Published:** 2019-08-06

**Authors:** Tamar Sofer, Leslie Emery, Deepti Jain, Alicia M. Ellis, Cathy C. Laurie, Matthew A. Allison, Jiwon Lee, Nuzulul Kurniansyah, Kathleen F. Kerr, Hector M. González, Wassim Tarraf, Michael H. Criqui, Leslie A. Lange, Walter R. Palmas, Nora Franceschini, Christina L. Wassel

**Affiliations:** 1 Division of Sleep and Circadian Disorders, Brigham and Women’s Hospital, Boston, MA USA; 2 Division of Sleep Medicine, Harvard Medical School, Boston, MA USA; 3 Department of Biostatistics, University of Washington, Seattle, WA USA; 4 Duke Clinical Research Institute, Duke University, Durham, NC USA; 5 Department of Family Medicine and Public Health, University of California – San Diego, La Jolla, CA USA; 6 Department of Neurosciences, Shiley-Marcos Alzheimer’s Disease Research Center, University of California San Diego, San Diego, CA United States of America; 7 Institute of Gerontology, Wayne State University, Detroit, MI USA; 8 Division of Biomedical Informatics and Personalized Medicine, University of Colorado-Denver, Denver, CO USA; 9 Department of Medicine, Columbia University, New York, NY USA; 10 Department of Epidemiology, University of North Carolina, Chapel Hill, NC USA; 11 Applied Sciences, Premier, Inc, Charlotte, NC USA

**Keywords:** Genome-wide association studies, Genetics research

## Abstract

Lower extremity peripheral artery disease (PAD) burden differs by race/ethnicity. Although familial aggregation and heritability studies suggest a genetic basis, little is known about the genetic susceptibility to PAD, especially in non-European descent populations. Genome-wide association studies (GWAS) of the ankle brachial index (ABI) and PAD (defined as an ABI < 0.90) have not been conducted in Hispanics/Latinos. We performed a GWAS of PAD and the ABI in 7,589 participants aged >45 years from the Hispanic Community Health Study/Study of Latinos (HCHS/SOL). We also performed GWAS for ABI stratified by Hispanic/Latino ethnic subgroups: Central American, Mexican, and South American (Mainland group), and Cuban, Dominican, and Puerto Rican (Caribbean group). We detected two genome-wide significant associations for the ABI in *COMMD10* in Puerto Ricans, and at *SYBU* in the Caribbean group. The lead SNP rs4466200 in the *COMMD10* gene had a replication p = 0.02 for the ABI in Multi-Ethnic Study of Atherosclerosis (MESA) African Americans, but it did not replicate in African Americans from the Cardiovascular Health Study (CHS). In a regional look-up, a nearby SNP rs12520838 had Bonferroni adjusted p = 0.05 (unadjusted p = 7.5 × 10^−5^) for PAD in MESA Hispanics. Among three suggestive associations (p < 10^−7^) in subgroup-specific analyses, *DMD* on chromosome X, identified in Central Americans, replicated in MESA Hispanics (p = 2.2 × 10^−4^). None of the previously reported ABI and PAD associations in whites generalized to Hispanics/Latinos.

## Introduction

The ankle brachial index (ABI) is the ratio of systolic blood pressure in the ankle to the arm, and reflects the degree of atherosclerotic obstruction in the lower extremity arteries. The ABI has been the major clinical diagnostic criterion for peripheral artery disease (PAD) for more than 40 years^1^. PAD, defined either by ABI ≤ 0.90 or via other criteria, is associated with an increased risk for incident cardiovascular disease (CVD) events and mortality^2–6^. Even borderline ABI values (0.90–1.00) are associated with increased risk of mortality^7^, as well as mobility impairment, including inability to walk 1/4 mile or climb one flight of stairs, as well as inability to complete a 6 minute walk^7^. The burden of PAD is greater in African-Americans and Cuban Americans compared to non-Hispanic whites and other Hispanic groups such as Mexican Americans^8,9^. However, these race/ethnic differences have not been explained by traditional, genetic, or novel risk factors to date^10–12^. Although familial aggregation and heritability estimates suggest a significant genetic contribution, little is known about the genetic susceptibility to PAD in non-European populations^13–16^.

GWAS conducted in European ancestry populations have identified the 9p21 locus significantly associated with ABI^17^, although associations were attenuated and no longer genome-wide significant after accounting for coronary artery disease (CAD). Additionally, a variant in *TCF7L2* was significantly associated with ABI in a large-scale candidate gene (∼50 K SNPs) analysis of European ancestry (n = 21,000), but this association failed to replicate in independent samples^12^. No significant associations were observed in more than 7,000 African-Americans in a candidate gene array study^12^. In general, previous studies have been limited by discovery of loci that have lacked specificity for PAD (e.g. 9p21 is also associated with CHD), a lack of racial/ethnic diversity, or a lack of robust independent replication of initial findings. In particular, no previous studies have examined the genetic variants underlying the ABI and PAD in Hispanic/Latino populations.

In this study, we sought to identify novel loci associations with ABI and PAD in a large cohort of US Hispanics/Latinos from the Hispanic Community Health Study/Study of Latinos (HCHS/SOL) using GWAS, replicate novel associations in independent samples, and to study whether previously identified genetic variants in European ancestry generalize to Hispanics/Latinos. We also performed GWAS for ABI in subgroups based on participant background, using genetically estimated ancestry and self-reported ethnicity, while taking into account their genetic and environmental heterogeneity.

## Methods

### HCHS/SOL population

The HCHS/SOL^18,19^ is a community-based cohort study of 16,415 self-identified Hispanic/Latino individuals aged 18–74 years and selected from households in predefined census-block groups across four US field centers (Chicago, IL; Miami, FL; Bronx, NY; and San Diego, CA). The HCHS/SOL baseline clinical examination occurred between 2008 and 2011 and included comprehensive biological, behavioral, and sociodemographic assessments. This study was approved by the institutional review boards (IRBs) at each field center, where all participants gave written informed consent, and by the Non-Biomedical IRB at the University of North Carolina at Chapel Hill, to the HCHS/SOL Data Coordinating Center. See Supplementary Material Section 3 for a complete list of IRBs that approved this study. This research was performed in accordance with relevant guidelines and regulations. The current study included participants who were aged >45 with measured ABI, and who had given DNA consent.

The cohort includes participants who self-identified as having a Hispanic/Latino background. The largest subgroups are Central American, Mexican, South American (comprising the Mainland group), Cuban, Dominican, and Puerto Rican (comprising the Caribbean group). Genetic analysis groups were based on these self-identified subgroups and on genetic similarity between individuals, as described in detail in Conomos, *et al*.^20^. In brief, clusters of individuals in the genetic principal components space were constructed while accounting for self-identified group. Therefore, these subgroups mostly overlap with self-reported background, while recovering classification in some individuals who did not report ethnicity, and in a few instances, assigning individuals who self-reported to be from one subgroup, to a different one^20^. Thus, we used these genetic analysis subgroups consequently, rather than self-reported background.

### Assessment of ABI and outcomes definitions

For each of the left and right side, the ABI was calculated as the maximum systolic blood pressure in the posterior tibial artery or the dorsalis pedis artery in the same leg, divided by the maximum systolic blood pressures in the left and right brachial arteries. The overall composite ABI was then calculated for each participant as the minimum of the left and right side ABI. A participant was classified as having PAD if the overall ABI was ≤0.90. We also defined a “borderline PAD” as having either ABI ≤1.00. These classifications are visualized in Fig. S1 in the Supplementary Information.

### Inclusion and exclusions criteria and study sample

The study sample included individuals aged 45–74 years (N = 7,662 genotyped individuals). Further exclusions were: missing ABI components of blood pressure data (N = 60), and primarily Asian genetic ancestry (n = 13, genetic outliers^20^), for a total of 7,589 individuals included in the primary ABI analysis. In the stratified analysis by Hispanic/Latino background group, additional 20 participants were excluded due to missing group, so final sample size numbers were: Central American (n = 760), Cuban (n = 1,500), Dominican (n = 700), Mexican (n = 2,619), Puerto Rican (n = 1,437), and South American (n = 553). For PAD and borderline PAD analyses, we excluded 192 individuals with arterial stiffness (ABI > 1.4), for N = 7,397 individuals included.

### Genotyping and imputation

HCHS/SOL individuals who consented for participation in genetic studies were genotyped using a HCHS/SOL custom 15041502 B3 array at Illumina. This array consisted of the Illumina Omni 2.5 M array (HumanOmni2.5-8 v.1-1), and additional ~150,000 SNPs selected based on multiple 1000 Genomes^21^ phase 1 data groups (CLM: Colombian in Medellin, Colombia); MXL: Mexican Ancestry in Los Angeles, California; and PUR: Puerto Rican in Puerto Rico), to increase the captured Amerindian genetic variation^22^. We applied standardized quality-assurance and quality-control (QA/QC) methods^23^ to generate recommended SNP- and sample-level quality filters, as previously described in Conomos, *et al*.^20^, who also provide comprehensive details of imputation. Following filtering for quality and informativeness (polymorphic and unduplicated), 2,232,944 SNPs were carried forward for imputation and downstream association analyses. Imputation was performed using the complete 1000 Genome phase 1 reference panel (n = 1,092)^21^. Genotypes were first pre-phased with SHAPEIT2 (v.2.r644)^24^ and then imputed with IMPUTE2 (v.2.3.0)^25^. Only variants with at least two copies of the minor allele present in any of the four 1000 Genomes continental panels were imputed. We performed downstream association analyses on the results 27,887,661 variants, and considered only variants with imputation quality oevar >0.3 and MAF ≥1%.

### Association testing

Since ABI had a highly skewed distribution (Fig. S1), and to prevent spurious associations, we winsorized ABI values so that the highest 1% of the values of ABI were set to the value of the 99th percentile of the distribution, and the lowest 1% of the values were set to the value of the 1st percentile of the distribution. After winsorization, the distribution was approximately normal. As a sensitivity analysis, for the top results we also report results from analysis that removed individuals with ABI >1.4.

To examine the association between genetic variants and the ABI, we fit a linear mixed model adjusted for sex, age, study center, sampling weights (to prevent potential selection bias due to the study sampling scheme), and the five first principal components as fixed-effects. We also had random-effects corresponding to genetic relatedness, household, and block unit sharing. For the binary traits of PAD and borderline PAD, we used the GMMAT algorithm^21^, which calculates the score test for each genetic variant based on a logistic mixed model. We used the same fixed- and random-effects as in the analysis of ABI.

For ABI, we investigated association results for variants with MAF ≥ 0.01, and (if imputed) imputation quality oevar ≥0.3. For the binary traits of PAD and borderline PAD, we further restricted the association results to variants with effective number of counts of the minor alleles, defined as effN = N × MAF × (1 − MAF) × oevar of at least 50, in both cases and controls. For the X-chromosome association testing, we set the count of alleles to be either 0 or 2 in males, and we calculated the effect allele frequency by separately calculating the minor allele frequency in males to get p_m_ (here count of alleles was either 0 or 1), separately in females to get p_f_, and combining the two as (N_m_p_m_ + 2N_f_ p_f_)/(N_m_ + 2N_f_), where N_m_ and N_f_ are the number of males and female individuals with measured/imputed genotypes at the locus, respectively. Associations were genome-wide significant if their p-value < 5 × 10^−8^, and we also investigated suggestive associations, with p-value < 10^−7^. In addition, for an X-chromosome association, we performed analysis stratified by sex, to study whether it is evidently driven by one of the sexes, or both.

### Stratified analysis by hispanic/latino ethnic background

To study potential differences in genetic association patterns between and within subgroups, we performed a secondary analysis of ABI in which a GWAS was conducted separately in each of the genetic analysis subgroups. We then meta-analyzed the summary statistics from each subgroup into a Mainland group and a Caribbean group separately. We used a fixed-effects meta-analysis that accounts for the correlations between the groups, due to relatives and shared environment^26^, and provide the p-value from the Cochran’s Q test of heterogeneity of effect sizes across the genetic analysis groups. To limit the potential number of false positive associations with the increased of the multiple testing burden due to performing a large number of GWAS, in the stratified analyses we considered only results from common variants, defined as those with approximate effective count of the minor allele effN (defined above) of at least 250, where this threshold is defined separately in each of the subgroups, yielding potentially different number of SNPs in each group. For reporting subgroup results, we used the same genome-wide significance and suggestive thresholds as in the main analysis.

### Generalization and replication analysis of discovered associations

To investigate the replication and generalization of findings from the stratified analysis to other populations, we tested the association of the index SNPs (SNPs with lowest p-value in a detected association region) with the ABI in Multi-Ethnic Study of Atherosclerosis (MESA)^27^ African Americans (N = 1,613), Hispanics/Latinos (N = 1,447), and European Americans (N = 2,527). In the Hispanic subgroup, 54% self-reported as Mexican descent, 12% as Dominican, 13% as Puerto Rican, 4% as Cuban, and 14% as Other Hispanic (primarily Central and South American groups), with 3% not reporting. Briefly, the MESA is a prospective population based study of European descent, African- American, Hispanic and Asian men and women aged 45–84 at the baseline examination in 2000–02 and designed to study subclinical cardiovascular disease and its progression, and risk factors that predict progression to clinically overt cardiovascular disease^27^. MESA was approved by the IRBs at each field center, where all participants gave written informed consent, and by the Human Subjects Division at the University of Washington, Seattle, WA, to the MESA Data Coordinating Center. See Supplementary Material Section 3 for complete list of IRBs approving this study.

All analyses were adjusted for age, sex, and five principal components. Since we interrogated five variants in three ethnic subgroups (as one of the detected SNPs was not available in MESA), the p-value criterion for replication was 0.05/15 = 0.003. Because it was previously shown to have better control of type 1 error and higher power, under the assumption that an association replicates only if the direction of estimated association matches between the discovery and replication study, we used one-sided p-values for replication testing^28^, with the expected direction of association in MESA depending on the direction of association in the HCHS/SOL. Therefore, if an association had the same direction in the HCHS/SOL and the replication study, the one-sided p-value is related to the usual two- sided p-value by p/2. However, if the directions of estimated associations do not match, the one-sided p-value is (1 − p/2), where p is the two-sided p-value.

Additional replication tests were performed in the Cardiovascular Health Study (CHS) and the Atherosclerosis Risk In Communities (ARIC), in almost 6,000 European Americans and 749 African Americans using genotypes and phenotypes downloaded from dbGaP. See Section 1.8 in the Supplementary Information for description.

As an exploratory analysis, and due to the high correlation between the traits and the observed heterogeneity in genetic association patterns, we defined regions of 10^5^ bp upstream and downstream of our top detected loci, and recorded the lead associated SNP for each of the ABI, PAD, and borderline PAD in each of the MESA replicating populations in these loci. The SNPs in these loci were restricted to those with MAF ≥ 0.05 and an imputation quality score of at least 0.8.

## Results

Study population characteristics overall and by self-reported Hispanic ethnic subgroup are provided in Table 1. There were 7,589 individuals in the primary ABI analysis. In the PAD analysis, there were 382 affected individuals with PAD and 7,015 unaffected (non-PAD without arterial stiffness). In the borderline PAD, there were 2,104 affected individuals and 5,293 unaffected (non-PAD/borderline PAD, and without arterial stiffness). ABI distribution varied in ethnic subgroups with the lowest mean among Cuban Americans, and highest mean among Mexican Americans.

**Table 1:** Participant Characteristics Overall and by Hispanic Ethnic Subgroup*.

	Overall n = 7589	Central American n = 760	South American n = 553	Mexican n = 2619	Puerto Rican n = 1437	Cuban n = 1500	Dominican n = 700
Age	55 (7)	55 (7)	55 (7)	55 (7)	56 (8)	56 (8)	55 (7)
Ankle Brachial Index	1.06 (0.13)	1.06 (0.13)	1.07 (0.12)	1.08 (0.12)	1.07 (0.15)	1.04 (0.12)	1.06 (0.13)
Male Sex	2966 (39%)	265 (35%)	207 (37%)	963 (37%)	575 (40%)	709 (47%)	242 (35%)
Systolic BP	132 (21)	132 (22)	128 (21)	127 (20)	135 (21)	135 (20)	136 (21)
Diastolic BP	78 (12)	78 (11)	75 (12)	75 (11)	79 (12)	80 (12)	81 (12)
Hypertension	3075 (41%)	295 (39%)	165 (30%)	866 (33%)	702 (49%)	715 (48%)	323 (46%)
Body Mass Index	30 (6)	31 (6)	29 (5)	30 (6)	31 (6)	29 (5)	30 (5)
Current smoker	1495 (20%)	89 (12%)	78 (14%)	381 (15%)	417 (29%)	467 (31%)	62 (9%)
Diabetes	2162 (28%)	214 (28%)	103 (19%)	784 (30%)	502 (35%)	361 (24%)	192 (27%)

*Mean +/− SD or n (%).

### Heritability estimation

We estimated the heritability of ABI using Haseman-Elston regression (^29^; Supplemental Information Section 1.3). The estimated heritability of ABI was 10% (95% CI: 3% to 18%) when including related individuals. The estimated contribution of the environmental factors household and block unit to the variance of ABI was 1% (95% CI: 0% to 6%). In the analysis including only unrelated individuals (n = 6,856), the estimated heritability was lower, 7%, (95% CI: 0% to 16%). The environmental contribution to the ABI variance had similarly a low estimate, with a 95% CI including zero.

### Overall pooled analysis of Hispanics/Latinos

GWAS inflation factors λgc^30^, were all between 0.99 and 1.005, indicating good control of population stratification. Manhattan plots and qq-plots for overall pooled analyses are provided in the Supplementary Figs S2 and S3. When examining the whole cohort, none of the variants were significantly associated with ABI, PAD, or borderline PAD at the genome-wide significance level.

### Stratified ABI analyses by Hispanic/Latino background

**Figure 1:**
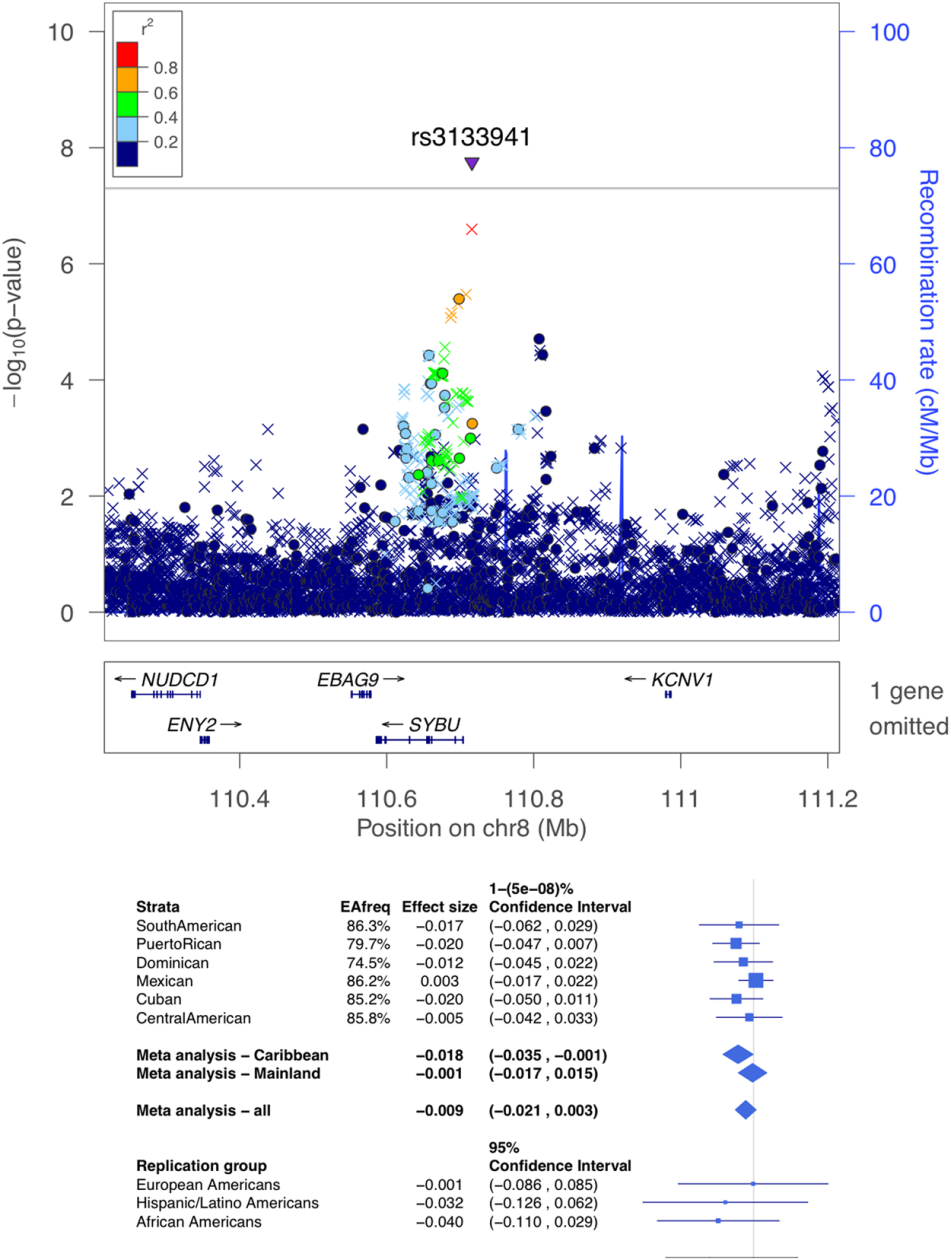
LocusZoom plot (top) and forest plot (bottom) of the *SYBU* locus, detected as associated with the ankle brachial index (ABI) in the Caribbean group. In the locusZoom plots, each point represents a variant, with location marked on the x-axis, and p-values marked as the location on the y-axis. The lead SNP is represented by the triangle, indicating that it is imputed. The colors of the variants correspond to the strength of their LD (r^2^) with the lead SNP, with LD estimated using the combined population of the HCHS/SOL Caribbean group. Circles correspond to genotyped variants, x symbols to imputed variants. The p-value of heterogeneity (across all HCHS/SOL genetic analysis groups) was 9.3 × 10^−4^. The bottom of the forest plot provides results from MESA replication groups.

**Figure 2:**
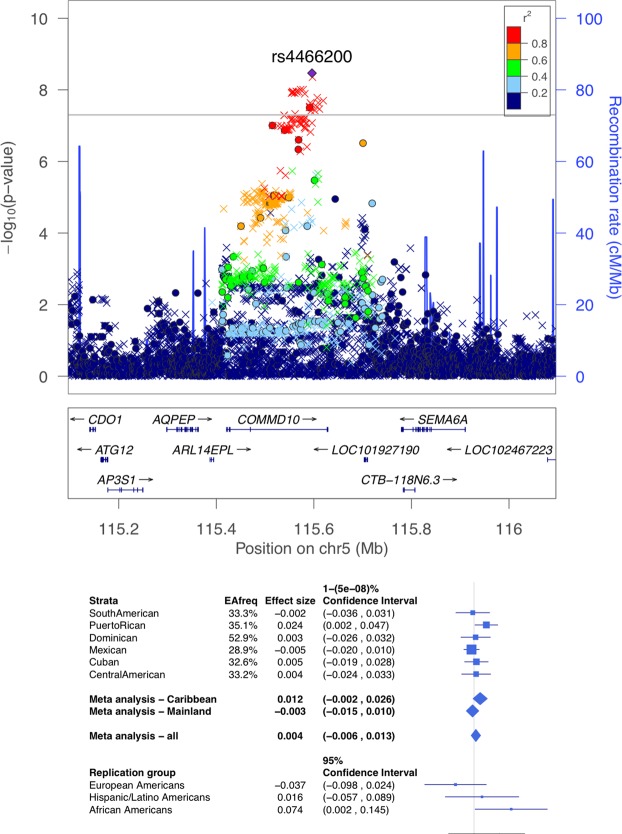
LocusZoom plot (top) and forest plot (bottom) of the *COMMD10* locus, detected as associated with ankle brachial index (ABI) in the Puerto Rican subgroup. In the locusZoom plot, each point represents a variant, with location marked on the x-axis, and p-values marked as the location on the y-axis. The lead SNP is represented by the diamond, indicating that it is genotyped. The colors of the variants correspond to the strength of their LD (r^2^) with the lead SNP, with LD estimated using the Puerto Rican population of the HCHS/SOL. Circles correspond to genotyped variants, x symbols to imputed variants. The p-value of heterogeneity (across all HCHS/SOL genetic analysis groups was 1 × 10^−10^. The bottom of the forest plot provides results from MESA replication groups.

Supplemental Fig. S4 displays the Manhattan plots the ABI stratified by Hispanic background subgroup. Table 2 provides the lead variants from six loci with p < 10^−7^ in subgroup stratified analyses for the ABI. Figures S8–S13 in the Supplementary Information provide regional association plots for all background subgroups for the variants reported in Table 2. For all loci identified in the stratified analysis, the heterogeneity test p was < 10^−3^. Further, the power to detect these association in the pooled analysis at the genome-wide significance level, assuming that the effect size in the group in which they were originally detected was the same across the population, were all >0.99. Table S1 in the Supplementary Information presents the PAD results for the top ABI SNPs in the subgroup-stratified analyses. Table S2 presents the pooled results for the ABI and PAD for the top ABI SNPs in the subgroup-stratified analysis. There were two genome-wide significant associations identified for ABI. The first locus with index SNP rs3133941 (p = 1.8 × 10^−8^) in the *SYBU* region on chromosome 8 (Fig. 1), was identified in the meta-analysis of the three Caribbean subgroups: Cuban, Dominican, and Puerto Rican (n = 3,637, MAF ranging from 14% to 26%). The second locus, *COMMD10* on chromosome 5 (Fig. 2), was identified in the analysis of Puerto-Ricans only (n = 1,437), with the index SNP rs4466200 (p = 3.4 × 10^−9^, MAF = 0.35).

**Table 2:** Significant or Suggestive Loci for the Ankle Brachial Index in Stratified Subgroup Analyses*.

Subgroup	Index SNP	Chr	Position	Allele A	Allele B	Type	EAF	Beta	SE	*p*-value	Het *p*-value	Gene
Dominican	rs6750426	2	15131582	G	A	G	0.75	0.031	0.006	7.39E-08	1.44E-06	
Puerto Rican	rs4466200	5	115596152	G	A	G	0.35	0.024	0.004	3.40E-09	<1E-06	*COMMD*10
Puerto Rican	rs7755533	6	157626334	G	A	G	0.59	0.022	0.004	9.28E-08	6.67E-06	*TMEM*242
Cuban	rs113916643	7	68415037	GA	G	I	0.73	−0.024	0.004	6.47E-0 8	4.03E-05	
Central American	rs6631478	X	32092380	T	C	G	0.69	−0.024	0.004	7.02E-08	2.41E-05	*DMD*
Caribbean	rs3133941	8	110715932	A	G	I	0.74–0.86	−0.018	0.003	1.78E-08	5.24E-01	*SYBU*

*Significant or suggestive SNPs with p < 10^−7^ in any of the subgroup-specific analyses, or in the meta-analysis of the Caribbean and the Mainland subgroups. Positions are provided in genome build 37. Allele A is the effect allele, EAF is the effect allele frequency, and Beta is the estimated effect of an increase in the allele count/dosage of allele A. Type is G when the variant is genotyped, and I when it is imputed. Het *p*-value is the *p*-values from the Cochran’s test of heterogeneity, adapted to the settings where there are correlated individuals between strata.

**Figure 3:**
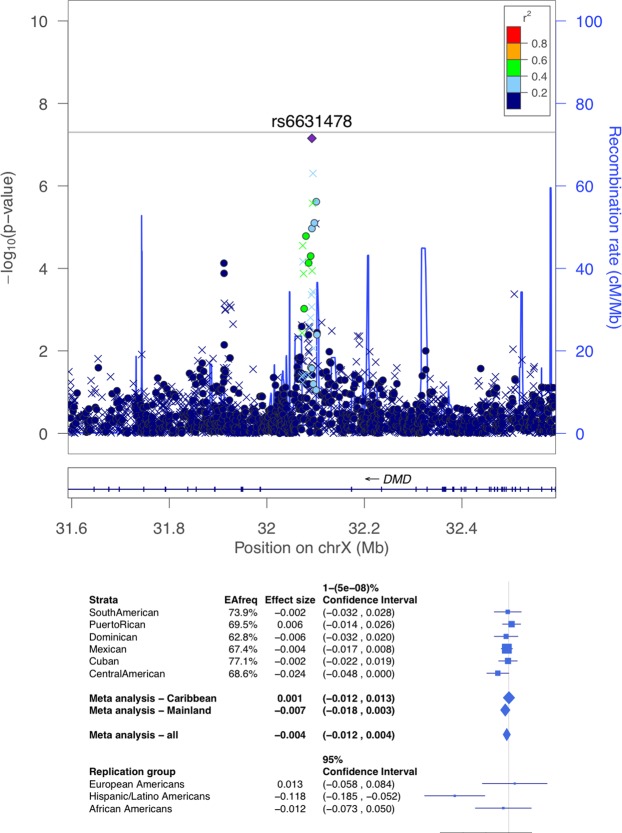
LocusZoom plot (top) and forest plot (bottom) of the *DMD* locus, detected as suggestively associated with ABI in the Central American genetic analysis group. In the locusZoom plots, each point represents a variant, with location marked on the x-axis, and p-values marked as the location on the y-axis. The lead SNP is represented by the diamond, indicating that it is genotyped. The colors of the variants correspond to the strength of their LD (r^2^) with the lead SNP, with LD estimated using the Central American population of the HCHS/SOL. Circles correspond to genotyped variants, x symbols to imputed variants. The p-value of heterogeneity (across all HCHS/SOL genetic analysis groups) was 2.41 × 10^−5^. The bottom of the forest plot provides results from MESA replication groups.

Among suggestive associations with the ABI (Supplemental Figs S5–S7, Fig. 3), rs6631478 in the *DMD* gene on the X-chromosome was identified in analysis of the Central American subgroup (Fig. 3; n = 760, p = 7.0 × 10^−8^, MAF = 0.31). A sex-stratified analysis at this locus suggested that the association exists in both sexes (males p = 4.2 × 10^−4^, females p = 1.2 × 10^−4^). There were three additional loci with p < 10^−7^, driven by associations with common variants, including an imputed indel, and two genotyped SNPs (Table 2).

To help contextualize these findings, Figs S18 and S19 in the Supplementary Information provide power analysis for the main (pooled) analysis, as well the group- and subgroup-specific GWAS, for a range of MAFs and effect sizes. One can see that the effect sizes required for the two genome-wide significant findings, given the analyses’ sample sizes and SNPs’ MAFs, are quite high: between 0.2–0.3 standard deviations (SDs) of ABI in the Caribbean group for the *SYBU* locus, and a little bit higher for the *COMMD10* locus detected in the Puerto-Ricans only. Indeed, the effect sizes reported for these loci in Table 2 are about 0.2 SDs of ABI (SDs of ABI in the HCHS/SOL range from 0.090 in Mexicans and South Americans to 0.105 in Puerto-Ricans). Sensitivity analysis removing a handful of individuals with ABI > 1.4 from the analysis reports similar results, as reported in Table S4 in the Supplementary Information.

### Replication and generalization results

Table 3 provides the replication results for both genome-wide significant and suggestive discovery results in the MESA cohort. Neither rs3133941 in *SYBU* nor rs4466200 in *COMMD10* replicated in MESA African American, European American or Hispanic groups, but rs4466200 had p = 0.02 in MESA African Americans (Table 3). However, this association did not replicate in African Americans from CHS (Table S5 in the Supplementary Information). Rs6631478 in the *DMD* gene replicated in MESA Hispanics, p = 2.2 × 10^−4^ (Table 3). Rs113916643 on chromosome 7 was not available in MESA. None of the associations replicated in European Americans (Table S6 in the Supplementary Information).

**Table 3:** Replication Results for Significant and Suggestive Loci for the ABI in Stratified Subgroup Analyses.

HCHS/SOL group	Index SNP	Chr	Allele A	Allele B	HCHS/SOL		MESA Hispanic		MESA AA		MESA EA	
					Beta	*p*-value	Beta	*p*-value	Beta	*p*-value	Beta	*p*-value
Dominican	rs6750426	2	G	A	0.031	7.39E-08	−0.006	5.58E-01	−0.019	6.79E-01	−0.023	7.93E-01
Puerto Rican	rs4466200	5	G	A	0.024	3.40E-09	0.016	3.34E-01	**0**.**074**	**2**.**16E**-**02**	−0.037	8.81E-01
Puerto Rican	rs7755533	6	G	A	0.022	9.28E-08	0.014	3.73E-01	0.024	2.82E-01	0.043	1.02E-01
Central American	rs6631478	X	T	C	−0.024	7.02E-08	−**0**.**118**	**2**.**22E**-**04**	−0.012	3.52E-01	0.013	6.43E-01
Caribbean	rs3133941	8	A	G	−0.018	1.78E-08	−0.032	2.50E-01	−0.04	1.27E-01	−0.001	4.94E-01

*Allele A is the effect allele. All variants were imputed, and all were common (MAF range 0.11–0.48 across all variants and all ethnic groups) in MESA. MESA replication sample sizes were n = 1,447 (Hispanic), n = 1,613 African American (AA), and n = 2,527 European American (EA). Rs113916643 was not available in MESA and is omitted from this table.

Supplementary Table S7 reports the results from the exploratory analysis that searched for the lead SNP in each of the MESA replicating populations and each of the traits. For the *DMD* locus identified for the ABI that replicated in MESA Hispanics, this analysis identified rs77460337, a SNP only 14 base-pairs away from the HCHS/SOL Central American lead SNP with a p = 5.3 × 10^−7^ in the PAD results of the MESA Hispanics. This SNP was not available in the HCHS/SOL data. At the *COMMD10* locus, rs12520838 was associated with PAD in MESA Hispanics (p = 7.5 × 10^−5^), a SNP that is 99,000 base-pairs away from the HCHS/SOL Puerto Rican lead variant (rs4466200); these variants are not in LD, as estimated in the HCHS/SOL.

None of the previously report associations with ABI or PAD in European descent, African-Americans or Japanese generalized to Hispanics/Latinos (all r-values = 1, see Supplementary Information Section 1.4 for methods and Supplementary Tables S8 and S9 for results). Additionally, Supplementary Figs S15 and S16 compare the effect sizes between the Hispanics/Latinos from our ABI analysis to the effect sizes in two previously published ABI analyses in individuals of European descent.

### SYTL3 gene region

A previous candidate gene study in European ancestry participants^16^ reported a significant association of rs2171209 in the *SYTL3* gene region on chromosome 6 with the ABI. Although this variant was not associated with ABI, PAD or borderline PAD in our analyses, we detected a suggestive association of rs317789, about 700 bp from rs2171209 and uncorrelated with rs317789, with borderline PAD (p = 7.56 × 10^−7^) in the overall pooled Hispanic/Latino HCHS/SOL analysis. To study whether this association is driven by the Mainland group (Central American, Mexican, South American), which has higher proportion of European ancestry on average, we tested the association of rs317789 with borderline PAD in the Mainland and Caribbean groups separately. However, there was no difference between the groups (Mainland p = 0.001, Caribbean p = 0.0002). Further, the association with the European descent index SNP rs2171209 was not significant in either the Mainland or Caribbean groups (p > 0.5 in both groups).

### Functional annotation of replicated association regions

At the *COMMD10* locus, the lead variant rs4466200 is in LD with 261 intronic variants (r2 ≥ 0.8) based in HCHS/SOL data, including three variants which have evidence for biological functionality. These include (1) rs12654321(r2 = 0.96) which lies in DNaseI hypersensitive genomic region and is bound by transcription factor CTCF in aortic adventitial fibroblast cells, (2) rs1382342, which is located in a DNaseI hypersensitive site in monocytes and inflammatory macrophage cells, and 3) rs4921067, which overlaps enhancer-binding transcription factor CEBPB binding site in IMR90 (normal human lung cell line) cells. Functional annotation in the heart tissues did not identify any strong candidates for likely casual regulatory variants at this locus. Most of the LD proxies are reported as eQTL for the *COMMD10* gene. At the chromosome X locus that replicated, the lead variant rs6631478 is located in the intronic region of the *DMD* gene and does not have any LD proxies (r^2^ ≥ 0.8 in HCHS/SOL data). Other significant and suggestive loci had no clear functional roles.

#### Colocalization analysis for the region around rs4466200

Using GTEx summary statistics we identified three genes in three tissues that had statistically significant eQTLs at the *COMMD10* gene region, and their list of significantly associated SNPs overlapped with the SNPs with p-value < 0.001 in the Puerto Rican ABI analysis. Sections 1.6 and 2.7 in the Supplementary Information provide complete description of the analysis and results, and Supplementary Table S10 in the Supplementary Information provides the results. There were four analyses, and two SNPs were identified. Rs4466200, the lead ABI Puerto Rican SNP had the highest posterior probability in the co-localization analysis with atrial appendage heart tissue, with the *COMMD10* gene, and in the analysis with the left ventricle heart tissue with the *SEMA6A* gene. However, these posterior probabilities were low: 0.08–0.09. An additional SNP in the region, rs4466200, also had higher posterior probabilities, although below our threshold of significance (0.75) – first, in the aortic artery tissue, with gene *CTB-118N6.3* (posterior probability 0.31), and second, in the left ventricle heart tissue with gene *COMMD10* (posterior probability 0.40).

#### Pathway enrichment analysis

We used GOrilla^31^ to search for enriched pathways based on gene scores from the pooled ABI analysis. Detailed methods are provided in Supplementary Information Section 1.7. After False Discovery Rate control (FDR), there were two pathways, both of type “function” (as annotated in GOrilla) that were significantly enriched (FDR q-value < 0.05): nucleosomal DNA binding (q-value = 0.002), and chromatin DNA binding (q-value = 0.002) pathways. Figure S17 in the Supplementary Information displays these pathways in a larger context of molecular function.

## Discussion

We examined the genetic architecture underlying ABI and PAD in the Hispanic/Latino population using a large cohort of ancestrally diverse Hispanics/Latinos living in the US. We report results from GWAS of ABI, PAD, and borderline PAD, both between and within ethnic subgroups based on genetic background. We identified two genome-wide significant loci (*SYBU, COMMD10*) and three suggestive associations for the ABI in analyses stratified by Hispanic/Latino ethnic background. However, we found no genome wide significant results in the overall pooled analyses of HCHS/SOL Hispanics/Latinos, perhaps due to significant genetic heterogeneity in risk for PAD among Hispanic/Latino subgroups^8^, as well as substantial differences in local and global genetic admixture among these groups, as suggested by differences in allele frequencies between groups in the detected association regions.

The *COMMD10* locus on chromosome 5 had p-value = 0.02 in MESA African-Americans for ABI, and regionally replicated for PAD in MESA Hispanics (FDR p = 0.05). This SNP had smaller estimated effect size in 749 African-Americans from CHS and replication p-value = 0.43. This could be because this is not a real signal, or due to low power, and difference in age distribution (CHS participants were 65 and older). *COMMD10* is a member of the COMM domain containing proteins, and encodes a protein that inhibits and mediates NF-κB, a transcription factor involved in innate and adaptive immune responses, and possibly with inflammatory processes^32,33^. Variants in *COMMD10* have been previously associated with multiple sclerosis^33^, inflammatory factors (tumor necrosis factor(TNF)-α and monocyte chemoattractant protein (MCP)-1)^34^, as well as chronic obstructive pulmonary disease (COPD) and asthma^35^. The functional annotation results showed *COMMD10* variants overlapping putative regulatory regions in adventitial fibroblast cells, inflammatory macrophages and monocytes, suggesting that the regulatory variants in *COMMD10* locus may be influencing the ABI or PAD via inflammatory and/or coagulation pathways, which is consistent with previously hypothesized roles for *COMMD10*. However, functional evaluation is needed to confirm the postulated underlying mechanisms. The co-localization analysis pointed to rs10062588 as a potential functional SNP in aortic artery and left ventricle heart tissues, however, the posterior probabilities were 0.31–0.40. This analysis is limited by the different populations between GTEx (European Ancestry) and HCHS/SOL, despite minor allele frequencies being quite similar (Supplementary Fig. S16).

The *DMD* locus on chromosome X replicated in MESA Hispanics for the ABI. Deletions, duplications, rearrangements, and point mutations in *DMD*, the dystrophin gene, have been strongly linked to Duchenne muscular dystrophy (DMD), Becker muscular dystrophy (BMD), and cardiomyopathy^36–39^. However, our functional annotation results did not identify any regulatory features associated with rs6631478.

None of the previously reported associations in populations of European, African, or East Asian (Japanese) ancestries generalized to Hispanics/Latinos, which could be due to the persisting low coverage of imputed genotypes using 1000 Genomes data, as well as the presence of genetic admixture. However, the stratified analysis revealed multiple associations with ABI, in specific Hispanic/Latino subgroups. These associations comprised largely common variants, and are therefore less likely to be statistical artifacts driven by outliers. In addition, these subgroup-specific associations were highly heterogeneous, as observed by comparing effect sizes and allele frequencies across Hispanic ethnic subgroups.

However, there is at least some evidence of common genetic basis at the regional level for ABI and PAD across race/ethnicities, with significant heterogeneity within each region. At the *SYTL3* locus, we identified a SNP associated with borderline PAD (rs317789, p < 10^−6^) in a close proximity (700 bp) to a previously reported association with the ABI in Europeans (rs2171209), although rs317789 is not correlated with rs2171209 in European or Hispanic populations. Variants in *SYTL3* have been previously associated with circulating lipoprotein(a) levels (Lp(a))^40^ which is a largely genetically regulated biomarker^41^. We have previously hypothesized that the association of *SYTL3* with the ABI and PAD may be mediated through Lp(a) levels^12^; however, circulating Lp(a) is unfortunately also not available in the HCHS/SOL to test this hypothesis. Our results for *SYTL3* also indicate that while the same loci may contribute to the underlying genetic architecture of PAD, heterogeneity within these loci and in ancestral background plays a substantial role.

This study has a few limitations. The ABI outcome is highly skewed, which may reduce power and increase type 1 error of genetic association studies^42^. Moreover, more than half of the study participants have ABI > 1. It is unclear which factors determine variation of ABI in the range of ABI > 1, and some of these factors are likely arterial elastic properties, height, and weight^43^. However, in a sensitivity analysis reported in Supplementary Table S3, we tested all associations reported in Table 2, with added adjustment to height and weight, and the results were essentially the same. For PAD analysis, the low number of cases of this population-based sample, reduces power, compared to a potential case-control study that would specifically target PAD cases. This may reduce the power of ABI analysis as well. Another limitation is the small sample size, resulting in low statistical power. The largest, Mexican subgroup, had 2,619 individuals, and the pooled analysis had 7,589 individuals, which is considered low for a GWAS. Future analyses should leverage the admixture pattern for genetic discoveries via admixture mapping, which has reduced multiple testing burden and was useful in other genetic analyses in this cohort^44–46^, and combine the HCHS/SOL GWAS with analyses in other cohorts in meta-analysis. Another limitation is the large number of tests, considering the different traits and subgroups: we performed ABI (pooled and by subgroup), PAD, and borderline PAD analyses. Still, the analyses were well controlled in terms of inflation, resulting in only a handful of findings among all analyses.

Additional evidence of regional heterogeneity in common genetic loci was observed in our MESA replication analysis. Specifically, an exploratory regional replication analysis of all common SNPs available in MESA around the HCHS/SOL lead SNPs from the stratified ABI analysis, detected two PAD SNPs different from the lead in HCHS/SOL: in the *DMD* locus, rs77460337 was 14 bp away from the HCHS/SOL Central American ABI lead SNP, and at the *COMMD10* locus, rs12520838 was 99,000 bp away from the HCHS/SOL Puerto Rican lead SNP. Overall, our generalization and replication results suggest there may be additional associations existing in both HCHS/SOL and MESA Hispanic populations, which may be detected once denser imputation panels are used, or whole genome sequencing data is available.

The current analysis detected multiple novel genetic variants associated with ABI in Hispanic/Latino ethnic subgroups from the HCHS/SOL. One of the lead SNPs in the *DMD* gene replicated in MESA Hispanics, despite the discovery being specific to Central Americans, and both the *DMD* and *COMMD10* loci regionally replicated in MESA Hispanics, albeit with different MESA-specific lead SNPs. Our results suggest regional commonality, but significant heterogeneity at the regional level for genetic architecture underlying the ABI and PAD. Care should be taken in interpreting these results due to the large multiple testing burden and low sample sizes within the Hispanic/Latino subgroups, and additional efforts for replication are needed, ideally, in diverse Hispanic/Latino populations. Another possible explanation for these results is that associated genotypes have different allele frequencies, and even different effect sizes, between ancestral populations (Europeans, Africans, Amerindians), and different admixture patterns in subpopulations of Hispanics/Latinos lead to different tag SNPs for the causal variants, and differences in power to discover these associations. Admixture mapping may help in revealing such insights. Future whole genome sequencing studies in diverse populations are important to facilitate better understanding and fine mapping of ABI/PAD loci, and may inform the mystery of lack of generalization of ABI/PAD loci across populations, including loci reported here, and in previous studies of European Ancestry and Japanese individuals.

## Supplementary information

Supplementary Information

## Data Availability

Genotype data of the HCHS/SOL and summary statistics from all discovery GWAS for ABI, PAD, borderline PAD, and arterial stiffness can be requested via dbGaP study accession phs000880. Complete meta-data related to these analyses is recorded for reproducibility in the HCHS/SOL Genetic Analysis Center’s Integrated Computing and Tracking system (unique analysis IDs are provided in Supplementary Table S11 in the Supplementary Information). Phenotype data can be requested via dbGaP study accession phs000810.
