# Supplementary material for: Variants Associated with the Ankle Brachial Index Differ by Hispanic/Latino Ethnic Group: a genome-wide association study in the Hispanic Community Health Study/Study of Latinos
Source: Sci Rep. 2019 Aug 6;9:11410. doi: 10.1038/s41598-019-47928-5 (PMC6684818; doi:10.1038/s41598-019-47928-5)
Supplement: Supplementary file 1 — Supplementary Information [file 41598_2019_47928_MOESM1_ESM.pdf]

Variants Associated with the Ankle Brachial Index Differ by Hispanic/Latino Ethnic Group: a genome-wide association study in the Hispanic Community Health Study/Study of Latinos: Supplementary Information

Tamar Sofer, Leslie Emery, Deepti Jain, Alicia M. Ellis, Cathy C. Laurie, Matthew A. Allison, Jiwon Lee, Nuzulul Kurniansyah, Kathleen F. Kerr, Hector M. González, Wassim Tarraf, Michael H. Criqui, Leslie A. Lange, Walter R. Palmas, Nora Franceschini*, Christina L. Wassel*

Contents

1 Supplemental Methods **2**

1.1 Classification of study individuals as having PAD and borderline PAD. 2

1.2 Genotyping, imputation, and quality control 4

1.3 SNP-Based Heritability Estimation of ABI 4

1.4 Generalization testing 5

1.5 Functional annotation 5

1.6 Colocalization analysis for high LD association regions 5

1.7 Pathway enrichment analysis 6

1.8 Replication testing in CHS and ARIC 7

2 Supplemental Results **7**

2.1 Manhattan and QQ plots from GWAS of ABI and PAD 7

2.2 Manhattan plots from stratified GWAS of ABI 9

2.3 LocusZoom and forest plots from suggestive ABI associations in HCHS/SOL genetic analysis groups 10

2.4 Additional results for top associations 14

*Equally contributed

2.5 Regional association figures (LocusZoom) for SNPs reported in Table 2, across Hispanic/Latino background subgroups 22
2.6 Generalization of known SNP-trait associations 29
2.7 Colocalization analysis of the region around rs4466200 with GTEx summary statistics . . 35
2.8 Pathway enrichment analysis 38

3 Power calculations 40

4 Institutional Review Board that approved this study 41

4.1 IRBs that approved the HCHS/SOL 41
4.2 IRBs that approved MESA 42

5 Reproducibility statement 42

1 Supplemental Methods

1.1 Classification of study individuals as having PAD and borderline PAD.

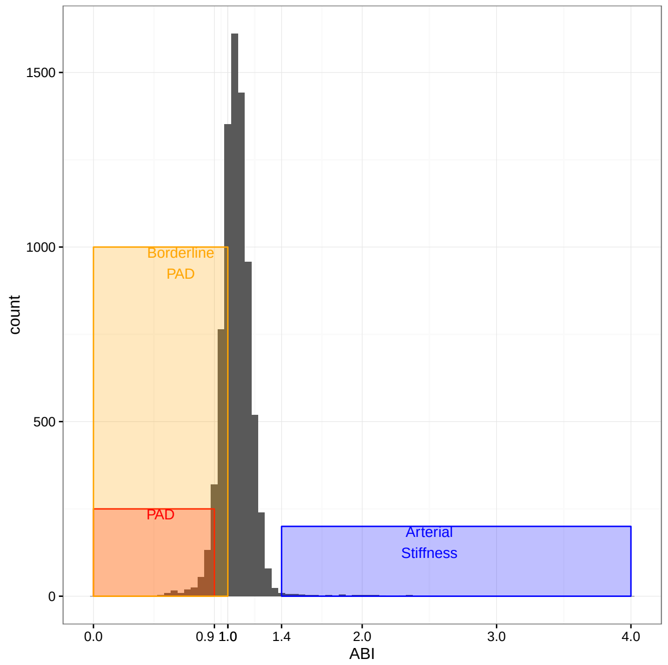

Figure S1: Classification of study individuals as having PAD and borderline PAD, based on their ABI measure. The figure shows the distribution of ABI in the 7,589 HCHS/SOL participants. An individual with ABI$\leq$ 0.9 was classified has having PAD (n=382), ABI$\leq$ 1 was classified has having borderline PAD (n=2,104 with PAD or borderline PAD). Also shown, ABI$>$ 1.4 defines arterial stiffness (n=192). The analyses of PAD and borderline PAD excluded individuals with arterial stiffness.

1.2 Genotyping, imputation, and quality control

Blood samples from HCHS/SOL individuals were genotyped the Illumina Omni 2.5M array which was customized to include an additional ∼150,000 markers selected as ancestry-informative markers, variants characteristic of Amerindian populations, known GWAS loci, and candidate gene polymorphisms.

Quality control was similar to the procedure described in Laurie et al. (2010) and included checks for sample identity, batch effects, missing call rate, chromosomal anomalies, deviation from Hardy-Weinberg equilibrium, Mendelian errors, and duplicate sample discordance. A total of 12,803 samples passed quality control, and 2,232,944 SNPs passed quality filters. Pairwise kinship coefficients and principal components reflecting ancestry were estimated using an iterative procedure which accounts for admixture (Conomos et al., 2016). Genome-wide imputation was performed using the 1000 Genomes Project phase 1 reference panel. Genotypes were first pre-phased with SHAPEIT2 (Delaneau et al., 2013) and then imputed with IMPUTE2 (Howie et al., 2009). Each imputed variant was assigned a quality score oevar, defined as the ratio between the observed variance and the expected variance of the allele count.

1.3 SNP-Based Heritability Estimation of ABI

We used Haseman-Elston regression to estimate the narrow-sense heritability of ABI and corresponding 95% confidence intervals (Sofer, 2017). ABI was regressed the same fixed effects as in the association analysis models, and the residuals from the main regression were regressed on the random effect matrices: kinship, household, and block unit. For this analysis, we used a kinship matrix estimated from all genotyped SNPs with MAF¿ 1% in the complete HCHS/SOL data set (without pruning by linkage disequilibrium (Conomos et al., 2016)). We used a model with all 7,589 individuals participating in the ABI analysis, and also a model without related individuals. For the latter, we randomly removed related individuals so that none of the pairwise estimated kinship coefficient was larger than $2^{-11/2}$ to obtain a set of 6,856 unrelated individuals (i.e. less than third degree relatives).

1.4 Generalization testing

We used the framework of Sofer et al. (2017) to study whether previously reported associations for ABI or PAD generalized to the HCHS/SOL. We took associations reported by each of the previous analyses, looked up the corresponding association results in the HCHS/SOL, and tested the generalization null hypotheses. The generalization null hypothesis was rejected if a SNP-trait association exists in both the discovery study and the HCHS/SOL, and the directions of association agree. For each tested association and direction, an FDR controlling r-value is computed, and generalization is declared if the r-value< 0.05. We considered previously discovered significant and suggestive variants for the ABI and PAD in individuals of European descent (Wassel et al., 2012; Kullo et al., 2014; Murabito et al., 2011), in African Americans (Wassel et al., 2012), and in Japanese (Koriyama et al., 2010; Matsukura et al., 2015).

1.5 Functional annotation

We conducted functional annotation to identify likely causal variants. At each significant locus, the lead and its correlated variants ($r^2 \geq 0.8$, calculated in the HCHS/SOL data) were interrogated against publicly available epigenomic datasets in the UCSC genome browser to determine if they overlapped with putative regulatory regions identified based on a) enrichment of histone modification ChIP-Seq (chromatin immunoprecipitation followed by sequencing) signal, example H3K4me1 and H3K27ac for enhancers, H3K4me3 for promoters b) DNaseI hypersensitivity and c) transcription factor binding. Since the identified significant loci could influence the phenotype via pathways of inflammation, coagulation, blood pressure regulation, and lipid, we included epigenomic datasets from heart, blood and adipose tissues for annotation available via the ENCODE (Consortium et al., 2012), BLUEPRINT (Adams et al., 2012), and Roadmap Epigenomics (Kundaje et al., 2015) Hubs in the UCSC genome browser (GRCh37/hg19 assembly, (Kent et al., 2002)). HaploReg (Ward and Kellis, 2011) was used to report the eQTL targets.

1.6 Colocalization analysis for high LD association regions

As a secondary analysis, we performed colocalization analysis using GTEx summary statistics version 7 (Carithers and Moore, 2015) with a detected association region that included more than 200 variants in

high LD with the lead SNP. We considered the six tissues that are relevant to ABI: whole blood, aorta, coronary artery, tibial artery, and, although less directly related, heart-atrial-appendage, and heart-left-ventricle, and all genes in a 1Mbp region centered at the lead SNP. We filtered SNPs by having ABI p-value< 0.001 in the analysis that identified the association, which was restricted to Puerto Ricans since this variant was significantly only in this subgroup. For GTEx data, for each tissue and gene, we first determined whether the gene was a significant eQTL (based on the summary statistics). If it was, we proceeded in the analysis, and considered SNPs that were statistically significant eQTLs for the tissue.

For each set of SNPs associated with a specific gene in a specific tissue that passed the filtering SNPs based on both ABI GWAS and the GTEx summary statistics, we used the coloc R (`https://cran.r-project.org/web/packages/coloc/index.html`) package to find the SNP with the highest posterior probability of being a causal SNP for both ABI and tissue-specific gene expression. This analysis was performed under the approximating assumption that the LD is the same for this SNPs in the Hispanic/Latino subgroup in which the ABI GWAS was performed, and in the GTEx donor population, which is primarily composed of individuals of European ancestry.

1.7 Pathway enrichment analysis

We performed a pathway enrichment analysis using GOrilla (Eden et al., 2009a, 2007). Then, we used Gorilla by providing the ordered list of gene, for all genes with p-value< 0.05 (1,106 genes), and the rest of the genes as a list of background genes. We considered enrichment in component, function, and process pathways.

P-value computation for genes. We first downloaded a gene range list from `https://www.cog-genomics.org/plink/1.9/resources`. For each gene in the list, we identified all genotyped SNP with minor allele count at least 250 in HCHS/SOL ABI pooled data set (we chose these criteria to limit the multiple testing burden while selecting only SNPs with high confidence in their testing results). We extracted the genotypes for these SNPs, and used the simpleM method (Gao et al., 2008, 2010) to compute the effective number of tests in the gene n_{eff}. Finally, we identified the smallest p-value p_{min} among the SNPs in the gene, and to account for multiple testing the final gene p-value was set to $\min\{1, p_{min} \times n_{eff}\}$.

1.8 Replication testing in CHS and ARIC

We downloaded genotype and phenotypes files for the ARIC (Atherosclerosis Risk In Communities; phs000280; phenotype files: pht004063 and topmed_dcc_demographic_v3.txt; ABI was the phenotype ABI04) and CHS (Cardiovascular Health Study; phs000287; phenotype file: pht001452; ABI was taken to be the minimum of RTAAI and LTAAI). For ARIC analysis we have 2,975 whites available ages 45-66, and for CHS, we had 3106 whites and 749 blacks ages 65-100. CHS and ARIC analyses were adjusted to age and sex, and accounted for genetic population structure using a Genetic Relationship Matrix (GRM) in a mixed model run using GEMMA (Zhou and Stephens, 2014). For these analyses, rather than winsorizing the ABI trait, as in the main HCHS/SOL analysis, we applied the fully-adjusted two-stage procedure that applies a rank-normalization distribution to the residuals of ABI after regression on covariates, and then use these as the outcome in an analysis, which again adjusts for the same covariates as the analysis that was used to obtain residuals (Sofer et al., 2019).

2 Supplemental Results

2.1 Manhattan and QQ plots from GWAS of ABI and PAD

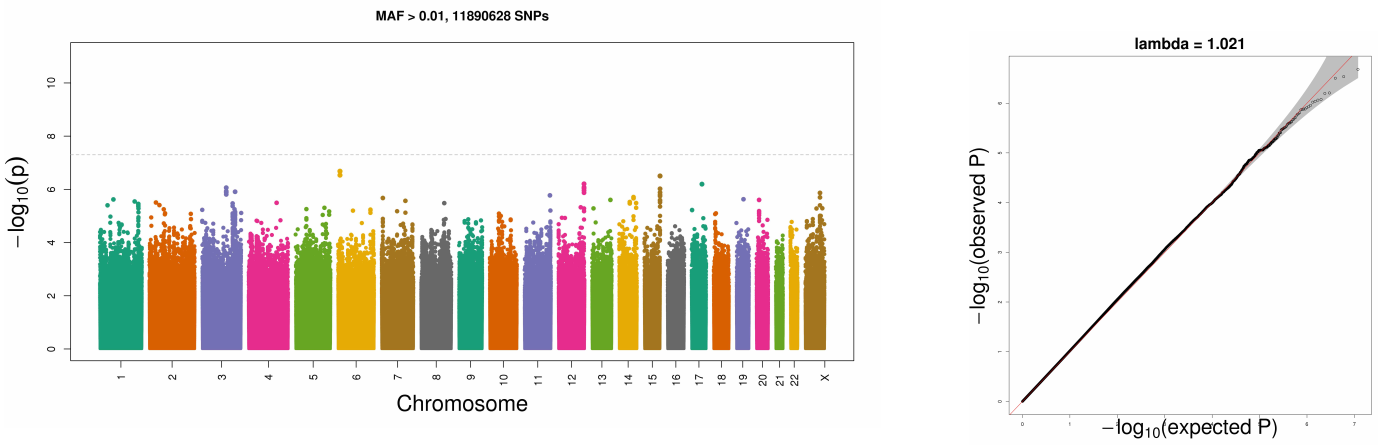

Figure S2: Manhattan and QQ plots of ABI GWAS SNPs were filtered by MAF> 0.1% and imputation quality oevar≥ 0.3.

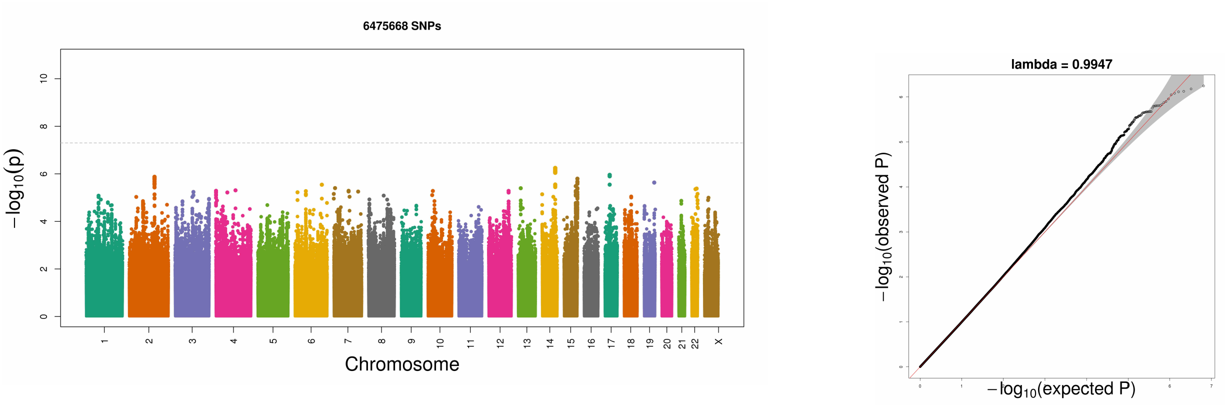

(a) PAD

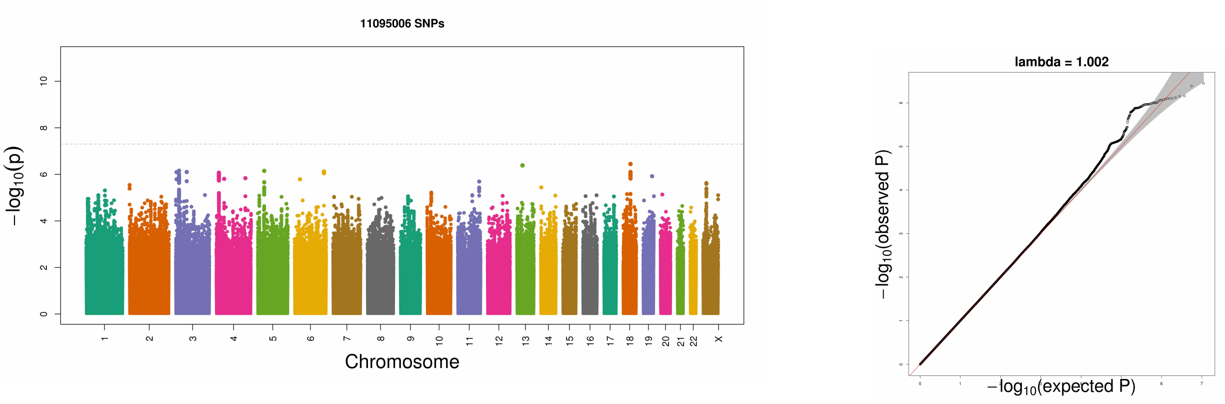

(b) Borderline PAD

Figure S3: Manhattan and QQ plots of PAD, and "borderline PAD" GWAS. SNPs were filtered by MAF $> 1\%$, imputation quality oevar≥ 0.3, as well as effective counts of the minor allele at least 50 in both cases and controls.

2.2 Manhattan plots from stratified GWAS of ABI

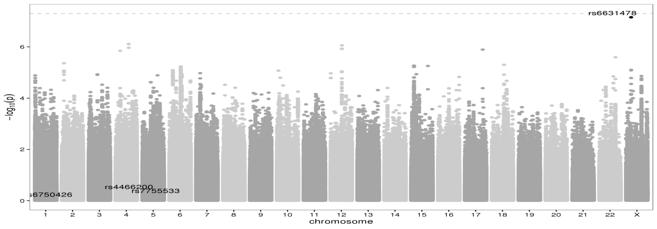

(a) Central American

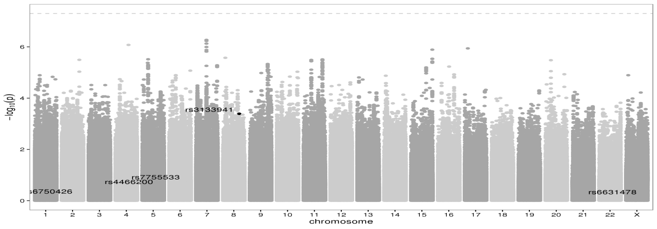

(b) Cuban

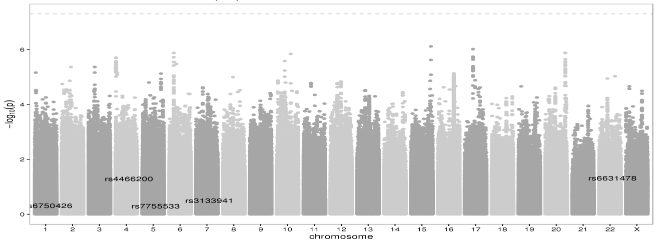

(c) Mexican

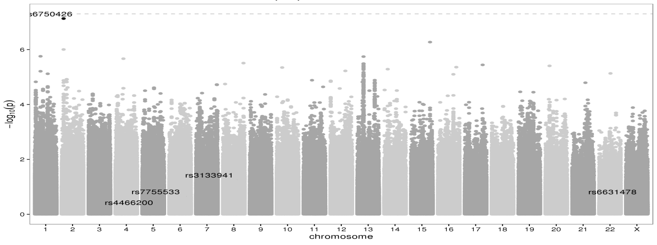

(d) Dominican

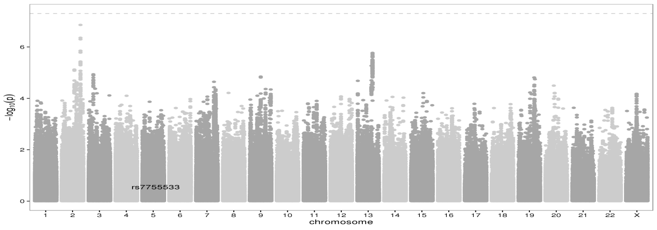

(e) South American

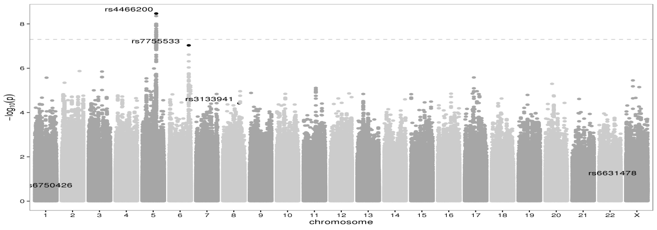

(f) Puerto Rican

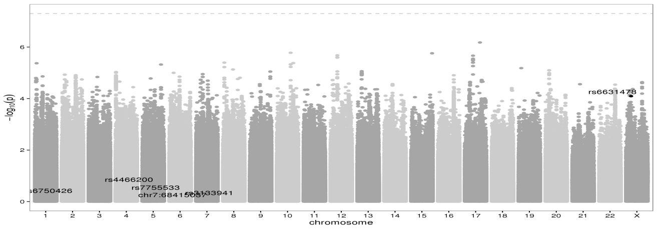

(g) Mainland meta-analysis

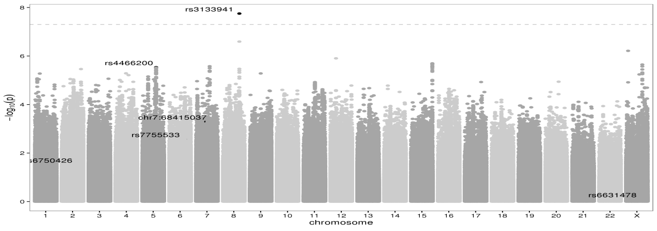

(h) Caribbean meta-analysis

Figure S4: Manhattan plots from the stratified analysis of ABI. On the left: the Mainland groups (meta-analysis at the bottom), on the right: the Caribbean groups (meta-analysis at the bottom). SNPs were filtered by effective count of the minor allele≥ 250.

2.3 LocusZoom and forest plots from suggestive ABI associations in HCHS/SOL genetic analysis groups

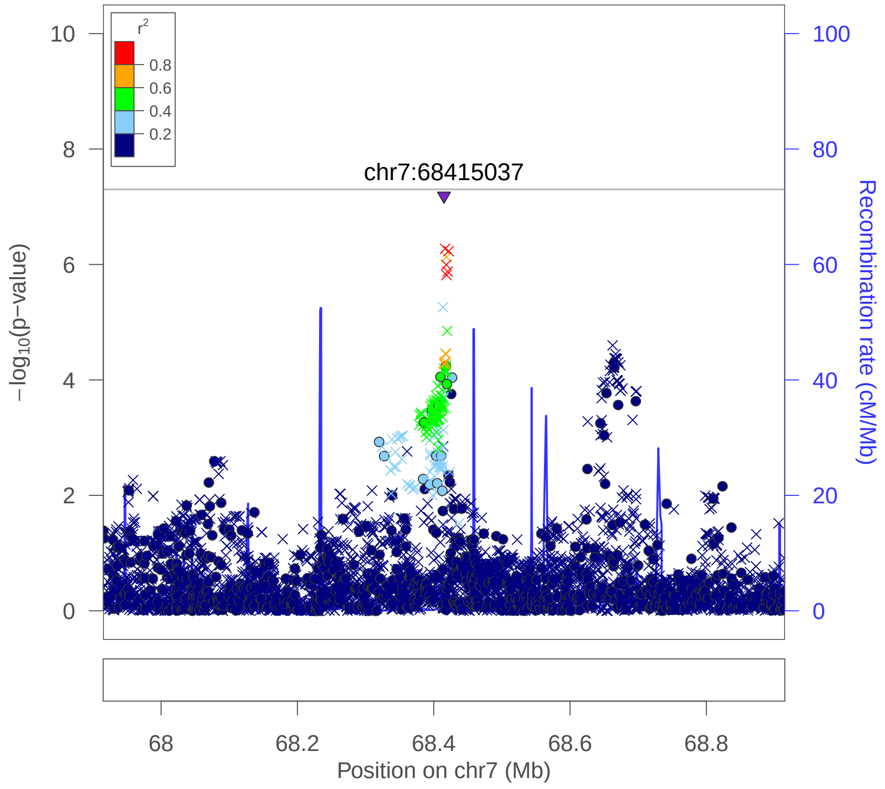

Strata	EAfreq	Effect size	1−(5e−08)% Confidence Interval
SouthAmerican	83.1%	−0.007	(−0.049 , 0.035)
PuertoRican	74.8%	0.003	(−0.023 , 0.028)
Dominican	81.4%	−0.005	(−0.042 , 0.032)
Mexican	78.9%	0.000	(−0.017 , 0.017)
Cuban	73.4%	−0.024	(−0.048 , 0.000)
CentralAmerican	80.4%	0.007	(−0.027 , 0.040)
**Meta analysis − Caribbean**		**−0.010**	**(−0.026 , 0.006)**
**Meta analysis − Mainland**		**0.001**	**(−0.014 , 0.015)**
**Meta analysis − all**		**−0.004**	**(−0.015 , 0.006)**

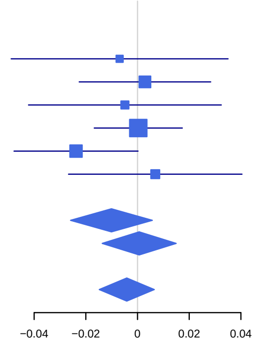

Figure S5: LocusZoom (top) plots and forest plot (bottom) of the chromosome 7 locus, detected as suggestively associated with ABI in the Cuban genetic analysis group. In the locusZoom plots, each point represent a variants, with location marked on the x-axis, and p-values marked as the location on the y-axis. The lead SNPs is represented by the triangle, indicating that it is imputed. The color of the variants correspond to the strength of their LD (R^2) with the lead SNP, with LD estimated using the Cuban population of the HCHS/SOL. Circles correspond to genotyped variants, x symbols to imputed ones. The p-value of heterogeneity (across all HCHS/SOL genetic analysis groups) was 4×10^{-5}.

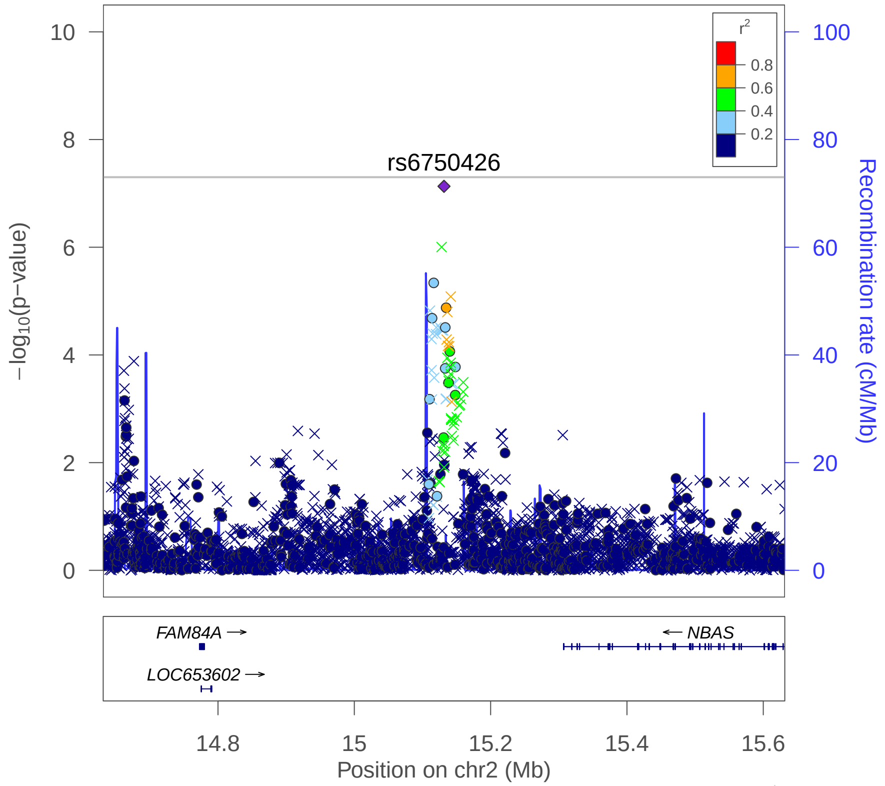

Strata	EAfreq	Effect size	1−(5e−08)% Confidence Interval
SouthAmerican	80.4%	0.016	(−0.022 , 0.055)
PuertoRican	69.1%	−0.005	(−0.028 , 0.019)
Dominican	74.6%	0.031	(−0.000 , 0.061)
Mexican	76.8%	−0.001	(−0.017 , 0.015)
Cuban	65.2%	0.002	(−0.020 , 0.024)
CentralAmerican	77.3%	0.001	(−0.030 , 0.033)
**Meta analysis − Caribbean**		**0.006**	**(−0.009 , 0.020)**
**Meta analysis − Mainland**		**0.001**	**(−0.012 , 0.015)**
**Meta analysis − all**		**0.003**	**(−0.006 , 0.013)**

Replication group	Effect size	95% Confidence Interval
European Americans	−0.023	(−0.079 , 0.032)
Hispanic/Latino Americans	−0.006	(−0.086 , 0.074)
African Americans	−0.019	(−0.097 , 0.060)

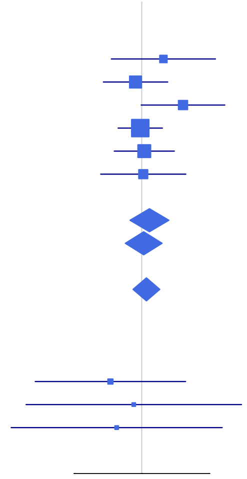

Figure S6: LocusZoom (top) plots and forest plot (bottom) of the chromosome 2 locus, detected as suggestively associated with ABI in the Dominican genetic analysis group. In the locusZoom plots, each point represent a variants, with location marked on the x-axis, and p-values marked as the location on the y-axis. The lead SNPs is represented by the diamond, indicating that it is genotyped. The color of the variants correspond to the strength of their LD (R^2) with the lead SNP, with LD estimated using the Dominican population of the HCHS/SOL. Circles correspond to genotyped variants, x symbols to imputed ones. The p-value of heterogeneity (across all HCHS/SOL genetic analysis groups) was 1.44×10^{-6}. The bottom of the forest plot provides results from MESA replication groups.

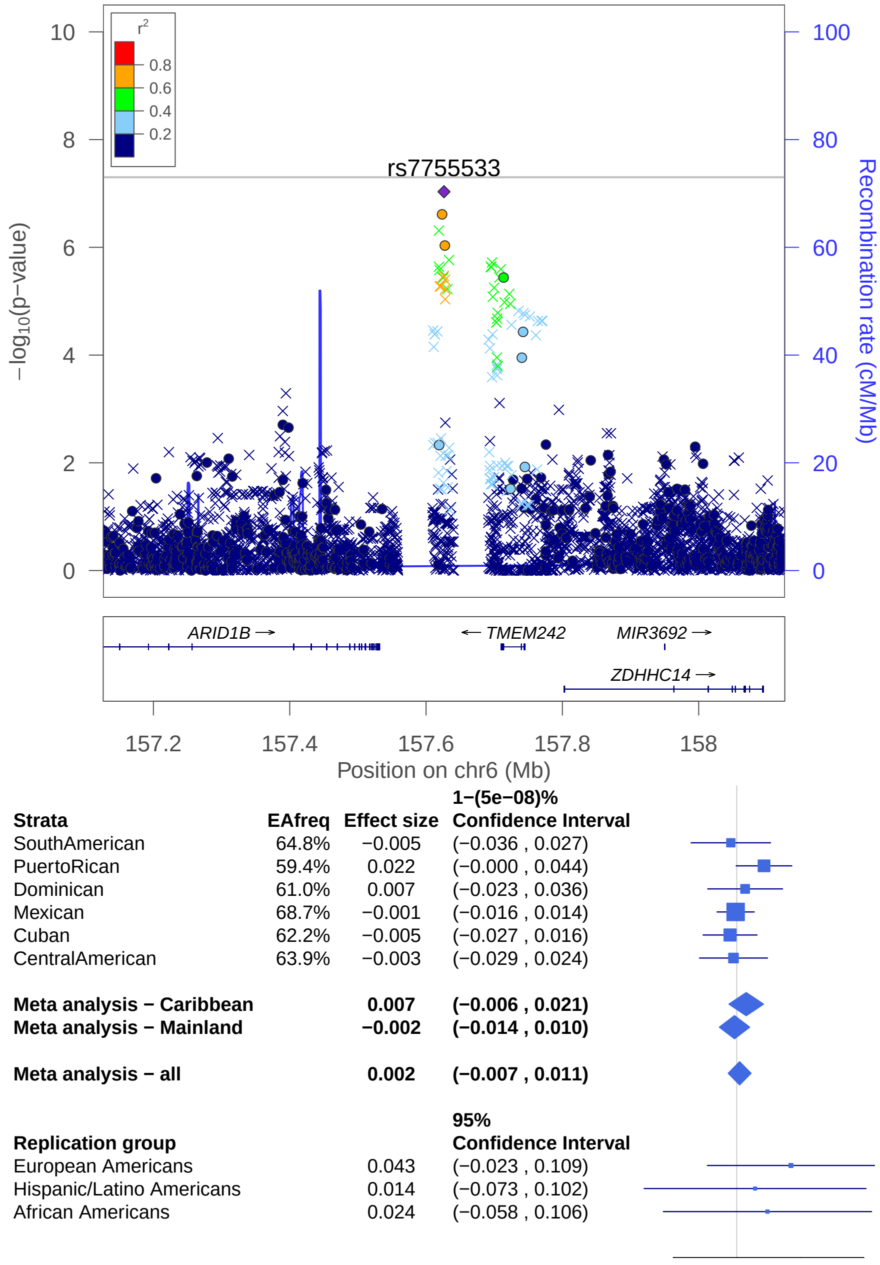

Figure S7: LocusZoom (top) plots and forest plot (bottom) of the $TMEM242$ locus, detected as suggestively associated with ABI in the Puerto Rican genetic analysis group. In the locusZoom plots, each point represent a variants, with location marked on the x-axis, and p-values marked as the location on the y-axis. The lead SNPs is represented by the diamond, indicating that it is genotyped. The color of the variants correspond to the strength of their LD (R^2) with the lead SNP, with LD estimated using the Puerto Rican population of the HCHS/SOL. Circles correspond to genotyped variants, x symbols to imputed ones. The p-value of heterogeneity (across all HCHS/SOL genetic analysis groups) was 6.7×10^{-6}. The bottom of the forest plot provides results from MESA replication groups.

2.4 Additional results for top associations

group	rsID	Chr	position	alleleA	alleleB	ABI Beta	ABI p-value	PAD direction	PAD p-value
Dominican	rs6750426	2	15131582	G	A	3.05E-02	7.39E-08	–	–
PuertoRican	rs4466200	5	115596152	G	A	2.45E-02	3.40E-09	< 1	5.85E-07
PuertoRican	rs7755533	6	157626334	G	A	2.15E-02	9.28E-08	< 1	7.49E-03
Cuban	rs113916643	7	68415037	GA	G	-2.38E-02	6.47E-08	> 1	6.48E-08
CentralAmerican	rs6631478	X	32092380	T	C	-2.39E-02	7.02E-08	> 1	2.48E-03
Caribbean	rs3133941	8	110715932	A	G	-1.78E-02	1.78E-08	> 1	3.34E-04

Table S1: Results from stratified association analysis of PAD with top results from stratified association analysis of ABI. Because we used a score test for PAD, results are given in terms of direction of association (< 1 and > 1 correspond to odds ratios smaller and higher than 1, respectively). Note that lower ABI correspond to PAD, so lower odds of PAD should give a higher (positive) effect size for ABI. Results for rs6750426 are not available because we required effN at least 50 in both cases and controls (see main manuscript, Association testing methods section), and this threshold was not met in the Dominican-only PAD analysis.

group	rsID	Chr	position	alleleA	alleleB	ABI beta in group	ABI p-value in group	ABI beta in pooled	ABI p-value in pooled	PAD direction in pooled	PAD p-value in pooled
Dominican	rs6750426	2	15131582	G	A	3.05E-02	7.39E-08	3.27E-03	7.26E-02	< 1	1.78E-01
PuertoRican	rs4466200	5	115596152	G	A	2.45E-02	3.40E-09	4.78E-03	5.74E-03	< 1	4.79E-02
PuertoRican	rs7755533	6	157626334	G	A	2.15E-02	9.28E-08	2.81E-03	8.97E-02	< 1	5.04E-01
Cuban		7	68415037	GA	G	-2.38E-02	6.47E-08	-4.60E-03	1.89E-02	> 1	3.47E-01
CentralAmerican	rs6631478	X	32092380	T	C	-2.39E-02	7.02E-08	-3.54E-03	1.66E-02	> 1	1.38E-01
Caribbean	rs3133941	8	110715932	A	G	-1.78E-02	1.78E-08	-1.01E-02	3.29E-06	> 1	2.71E-03

Table S2: Results from pooled (not stratified by background group) association analyses of ABI and PAD in the top results from stratified association analysis of ABI. Because we used a score test for PAD, results are given in terms of direction of association (< 1 and > 1 correspond to odds ratios smaller and higher than 1, respectively). Note that lower ABI correspond to PAD, so lower odds of PAD should give a higher (positive) effect size for ABI.

group	rsID	Chr	Position	AlleleA	AlleleB	Type	EAF	Beta	SE	p-value	Gene
Dominican	rs6750426	2	15131582	G	A	G	0.7394	0.03	0.006	6.37E-08	
Puerto Rican	rs4466200	5	115596152	G	A	G	0.3528	0.025	0.004	2.19E-09	COMMD10
Puerto Rican	rs7755533	6	157626334	G	A	G	0.5995	0.021	0.004	1.51E-07	TMEM242
Cuban	rs113916643	7	68415037	GA	G	I	0.7346	-0.022	0.004	4.51E-07	
Central American	rs6631478	X	32092380	T	C	G	0.6858	-0.023	0.004	2.35E-07	DMD
Caribbean	rs3133941	8	110715932	A	G	I	0.7407-0.8478	-0.018	0.003	1.63E-08	SYBU

Table S3: Results from sensitivity analysis that further adjusts for height and weight in addition to other covariates, applied to the SNPs reported in Table 2 of the main manuscript. Note that this analysis included 59 less people who withdrew consent for genetic studies by the time the revision for this manuscript was prepared, and were no longer available for analysis.

group	rsID	Chr	Position	AlleleA	AlleleB	Type	EAF	Beta	SE	p-value	Gene
Dominican	rs6750426	2	15131582	G	A	G	0.74	0.03	0.006	1.12E-07	
Puerto Rican	rs4466200	5	115596152	G	A	G	0.35	0.024	0.004	3.6E-09	COMMD10
Puerto Rican	rs7755533	6	157626334	G	A	G	0.60	0.021	0.004	1.01E-07	TMEM242
Cuban	rs113916643	7	68415037	GA	G	I	0.73	-0.023	0.004	1.24E-07	
Central American	rs6631478	X	32092380	T	C	G	0.69	-0.024	0.004	9.42E-8	DMD
Caribbean	rs3133941	8	110715932	A	G	I	0.74-0.85	-0.017	0.003	12.57E-08	SYBU

Table S4: Results from sensitivity analysis that removed individuals with ABI ¿ 1.4 from the ABI analysis, applied to the SNPs reported in Table 2 of the main manuscript. Exclusions based on ABI ¿1.4 included 3 Dominicans, 3 Puerto Ricans, 3 Cubans, 7 Central Americans, and overall 10 Caribbean individuals.

						MESA AA		CHS AA		Replication p-values		
Discovery group	rsID	Chr	Discovery Beta	alleleA	alleleB	Beta	p-value	Beta	p-value	MESA	CHS	Combined
PuertoRican	rs4466200	5	0.024	G	A	0.074	0.043	0.00198	0.87	0.02	0.43	0.06
PuertoRican	rs7755533	6	0.022	G	A	0.024	0.56	-0.00381	0.73	0.28	0.64	0.44
CentralAmerican	rs6631478	X	-0.024	T	C	-0.012	0.70	0.00713	0.41	0.35	0.79	0.62
Caribbean	rs3133941	8	-0.018	A	G	-0.04	0.25	-0.00459	0.67	0.13	0.33	0.13

Table S5: Results from replication analysis in MESA and CHS African Americans, for four of the loci provided in Table 2 in the main manuscript (one locus was not available in MESA, two were not available in CHS). Analysis for MESA used the same analytic plan as the one for HCHS/SOL. CHS analysis used rank-normalized transformed residuals. Replication p-values are one-sided p-values with direction guided by the direction of association ("Discovery Beta") in the HCHS/SOL. Replication p-values were combined using the sumz function in the R package metap (Dewey, 2019).

						MESA EA		CHS EA		ARIC EA		Replication p-values			
Discovery group	rsID	Chr	Discovery Beta	allele A	allele B	Beta	p-value	Beta	p-value	Beta	p-value	MESA	CHS	ARIC	Combined
PuertoRican	rs4466200	5	0.024	G	A	-0.037	0.24	0.006	0.14	-0.001	0.74	0.88	0.07	0.63	0.51
PuertoRican	rs7755533	6	0.022	G	A	0.043	0.2	-0.003	0.55	-0.002	0.57	0.1	0.73	0.72	0.48
CentralAmerican	rs6631478	X	-0.024	T	C	0.013	0.71	0.0004	0.93	0.004	0.26	0.64	0.54	0.87	0.82
Caribbean	rs3133941	8	-0.018	A	G	-0.0006	0.99	0.006	0.29	-0.0005	0.92	0.49	0.85	0.46	0.70

Table S6: Results from replication analysis in MESA, CHS, and ARIC European Americans, for four of the loci provided in Table 2 in the main manuscript (one locus was not available in MESA, two were not available in either CHS or ARIC). Analysis for MESA used the same analytic plan as the one for HCHS/SOL. CHS and ARIC analyses used rank-normalized transformed residuals. Replication p-values are one-sided p-values with direction guided by the direction of association ("Discovery Beta") in the HCHS/SOL. Replication p-values were combined using the sumz function in the R package metap (Dewey, 2019).

Table S7: Results from exploratory regional analysis in a 200,000 region centered around the lead SNPs in the stratified ABI analysis reported in Table 2 in the main manuscript. For each of the loci, traits, and MESA population, we report the lead MESA SNP in the region, the number of SNPs tested in the region, and the regional Bonferroni adjusted p-value, calculated as $\min(1, p\text{-value} * n_{\text{snp}})$ where n_{snp} is the number of SNPs in the region.

trait	MESA population	Chr	rsID	p-value	n SNPs in region	adjusted p-value
ABI	AA	2	rs1178200	5.93E-03	813	1.00E+00
PAD	AA	2	rs11903463	3.22E-03	813	1.00E+00
Borderline PAD	AA	2	rs10176960	1.85E-03	813	1.00E+00
ABI	EA	2	rs10186602	1.44E-02	600	1.00E+00
PAD	EA	2	rs115122011	2.32E-02	600	1.00E+00
Borderline PAD	EA	2	rs17433057	4.18E-03	600	1.00E+00
ABI	H/L	2	rs6432494	2.10E-03	700	1.00E+00
PAD	H/L	2	rs17433057	5.77E-04	700	4.04E-01
Borderline PAD	H/L	2	chr2:15063376:D	4.96E-02	700	1.00E+00
ABI	AA	5	rs1677405	5.29E-03	971	1.00E+00
PAD	AA	5	rs12109104	3.09E-03	971	1.00E+00
Borderline PAD	AA	5	rs11951083	1.01E-02	971	1.00E+00
ABI	EA	5	rs7721593	6.70E-02	740	1.00E+00
PAD	EA	5	rs73780900	1.49E-02	740	1.00E+00
Borderline PAD	EA	5	rs62385976	1.07E-02	740	1.00E+00
ABI	H/L	5	rs17139457	6.60E-02	715	1.00E+00
PAD	H/L	5	rs12520838	7.54E-05	715	5.39E-02
Borderline PAD	H/L	5	rs173816	7.30E-03	715	1.00E+00
ABI	AA	6	rs113373350	1.13E-02	46	5.21E-01
PAD	AA	6	rs151041972	8.38E-02	46	1.00E+00
Borderline PAD	AA	6	rs113373350	3.86E-02	46	1.00E+00
ABI	EA	6	rs142440005	7.25E-02	73	1.00E+00
PAD	EA	6	rs145981703	1.78E-02	73	1.00E+00
Borderline PAD	EA	6	rs7450640	4.76E-01	73	1.00E+00
ABI	H/L	6	rs28695567	4.54E-01	57	1.00E+00

Table S7: Results from exploratory regional analysis in a 200,000 region centered around the lead SNPs in the stratified ABI analysis reported in Table 2 in the main manuscript. For each of the loci, traits, and MESA population, we report the lead MESA SNP in the region, the number of SNPs tested in the region, and the regional Bonferroni adjusted p-value, calculated as $\min(1, p\text{-value} * n_{\text{snp}})$ where n_{snp} is the number of SNPs in the region.

trait	MESA population	Chr	rsID	p-value	n SNPs in region	adjusted p-value
PAD	H/L	6	rs28695567	2.31E-03	57	1.31E-01
Borderline PAD	H/L	6	rs28695567	2.43E-02	57	1.00E+00
ABI	AA	X	rs16990010	2.57E-02	214	1.00E+00
PAD	AA	X	rs6628670	1.55E-02	214	1.00E+00
Borderline PAD	AA	X	rs1921379	1.70E-02	214	1.00E+00
ABI	EA	X	rs1293908	4.11E-02	265	1.00E+00
PAD	EA	X	rs1966254	2.75E-03	265	7.29E-01
Borderline PAD	EA	X	rs113166270	5.33E-02	265	1.00E+00
ABI	H/L	X	rs6631478	4.45E-04	291	1.29E-01
PAD	H/L	X	rs77460337	5.33E-07	291	1.55E-04
Borderline PAD	H/L	X	rs77460337	3.52E-03	291	1.00E+00
ABI	AA	8	rs76336187	9.10E-03	669	1.00E+00
PAD	AA	8	rs77036455	7.98E-04	669	5.34E-01
Borderline PAD	AA	8	chr8:110800631:D	2.86E-03	669	1.00E+00
ABI	EA	8	chr8:110753742:D	2.21E-01	421	1.00E+00
PAD	EA	8	chr8:110628293:I	9.20E-03	421	1.00E+00
Borderline PAD	EA	8	rs13261623	1.56E-01	421	1.00E+00
ABI	H/L	8	rs7831266	4.01E-02	467	1.00E+00
PAD	H/L	8	chr8:110623678:I	1.13E-03	467	5.30E-01
Borderline PAD	H/L	8	rs10098812	2.51E-02	467	1.00E+00

2.5 Regional association figures (LocusZoom) for SNPs reported in Table 2, across Hispanic/Latino background subgroups

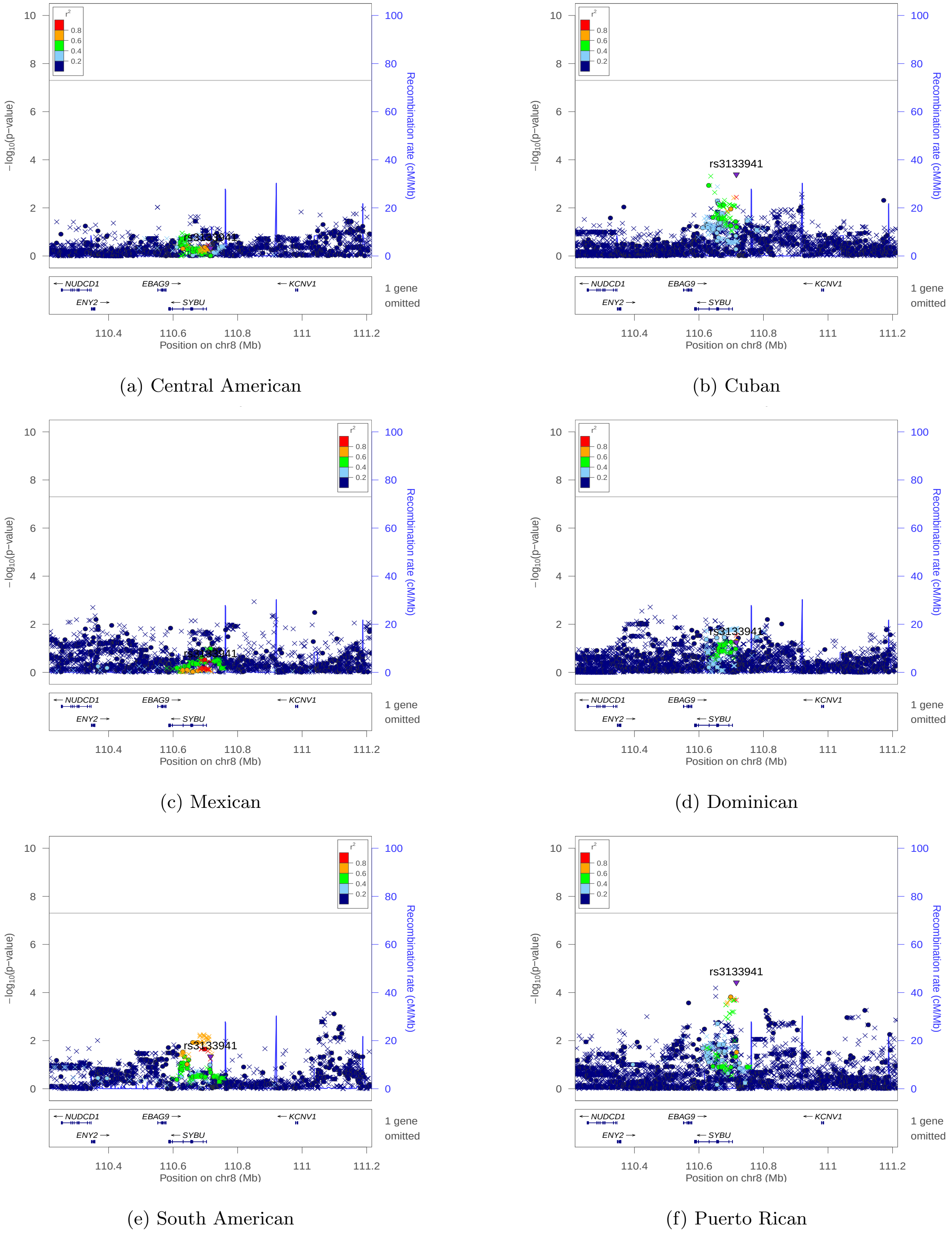

Figure S8: Regional association plots across the Hispanic/Latino background subgroups for SNP rs3133941, detected in the Caribbean group (right column).

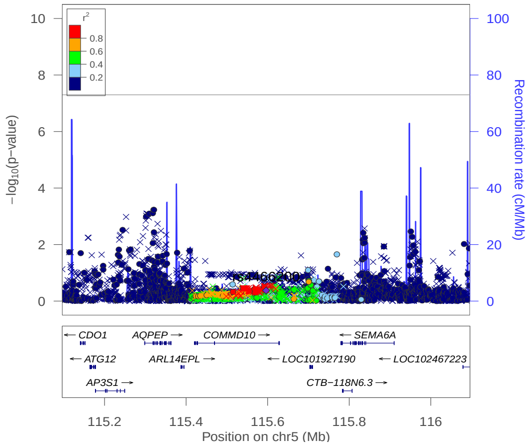

(a) Central American

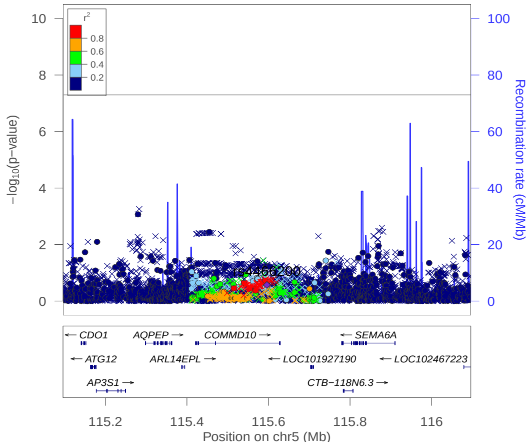

(b) Cuban

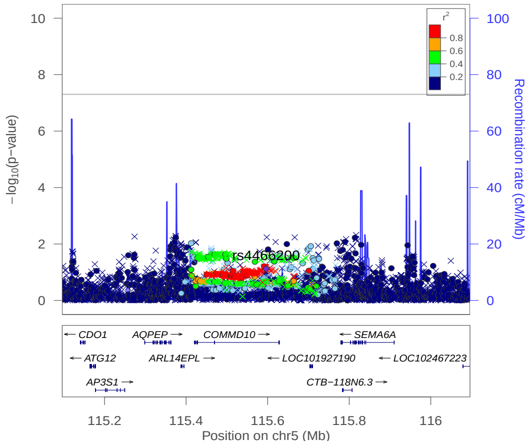

(c) Mexican

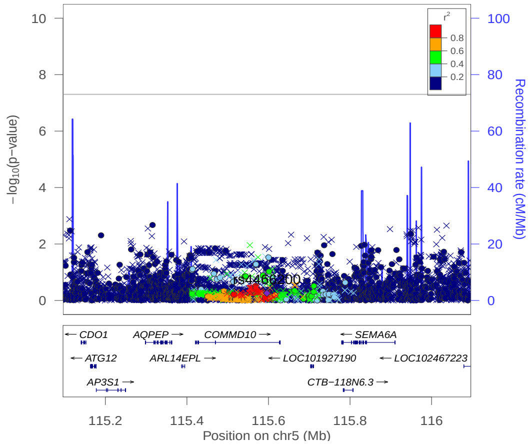

(d) Dominican

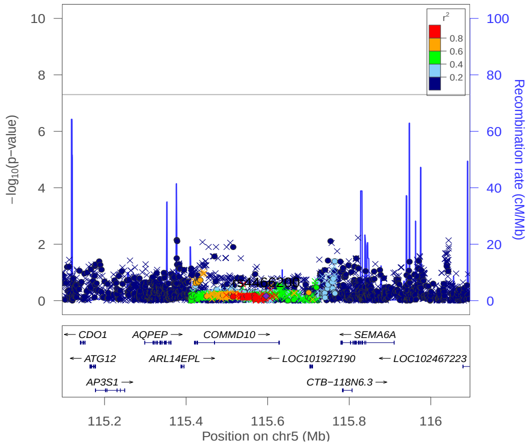

(e) South American

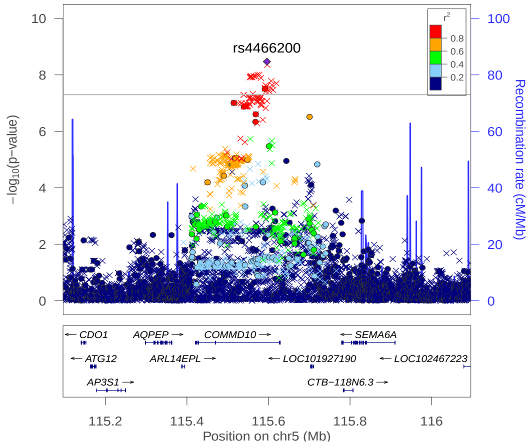

(f) Puerto Rican

Figure S9: Regional association plots across the Hispanic/Latino background subgroups for SNP rs4466200, detected in the Puerto Rican subgroup.

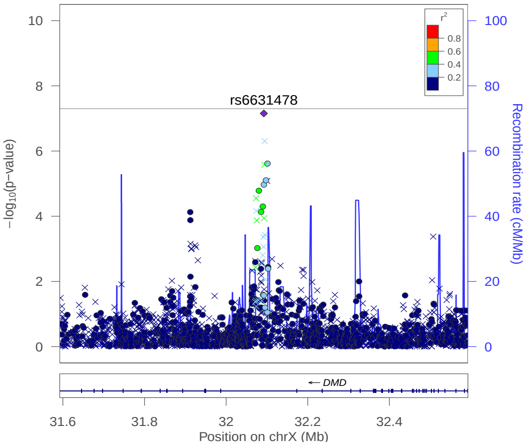

(a) Central American

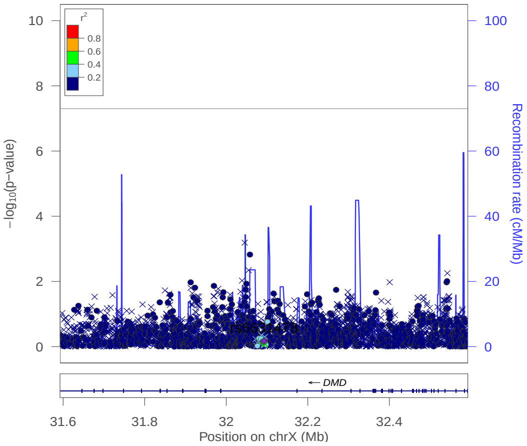

(b) Cuban

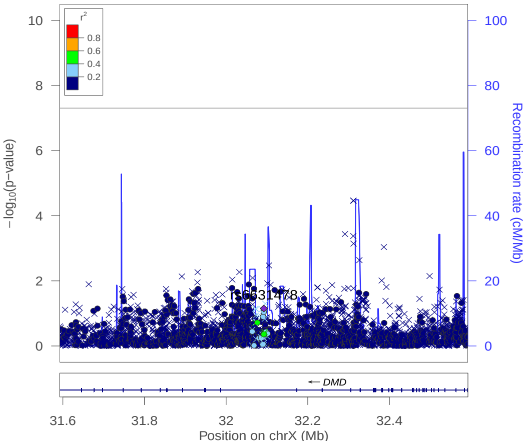

(c) Mexican

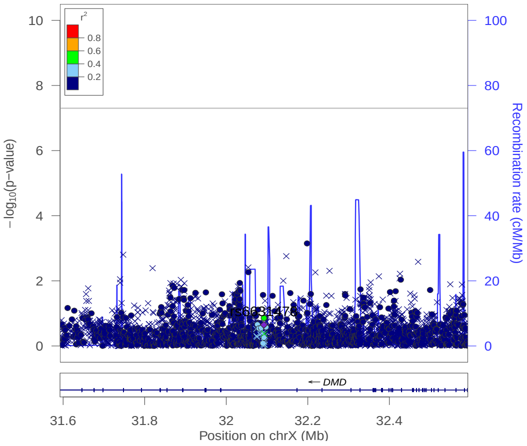

(d) Dominican

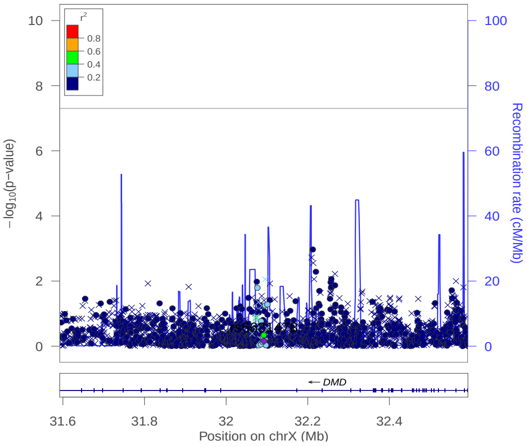

(e) South American

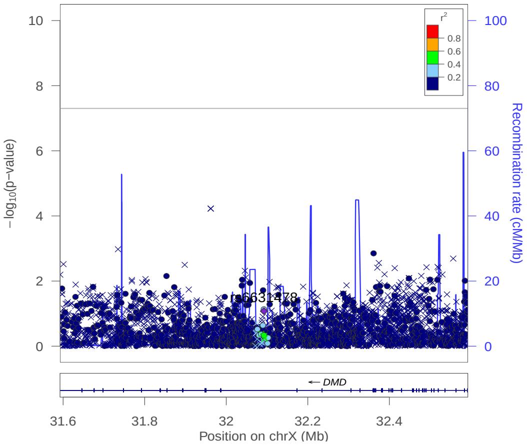

(f) Puerto Rican

Figure S10: Regional association plots across the Hispanic/Latino background subgroups for SNP rs6631478, detected in the Central American subgroup.

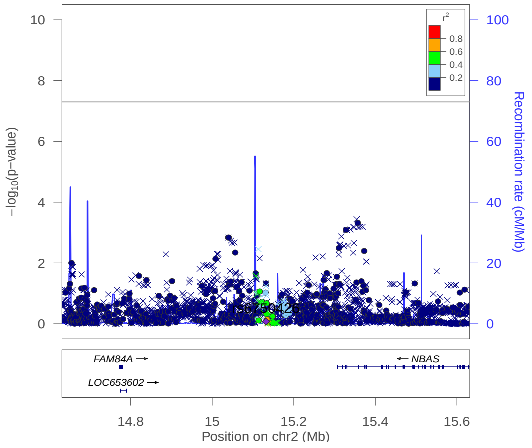

(a) Central American

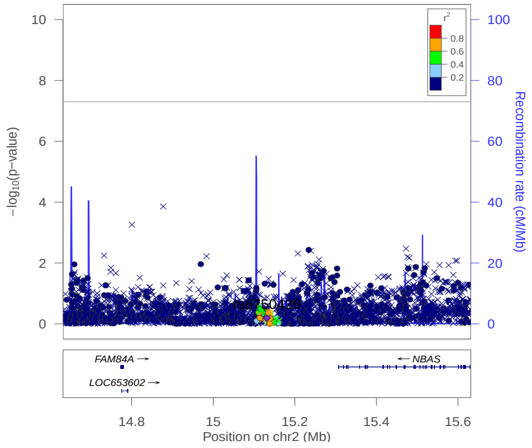

(b) Cuban

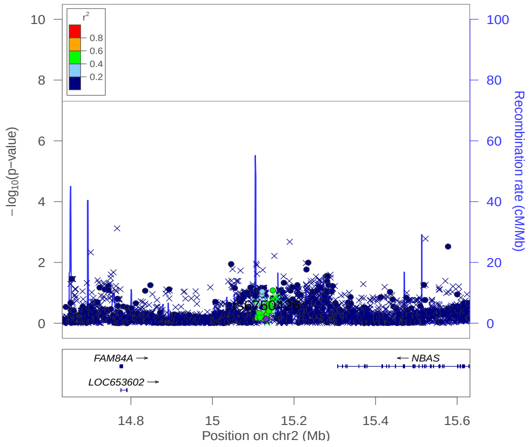

(c) Mexican

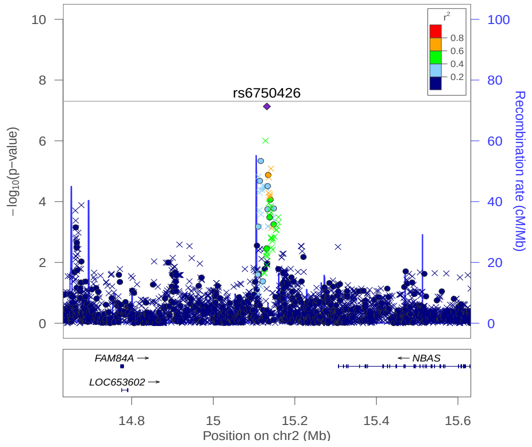

(d) Dominican

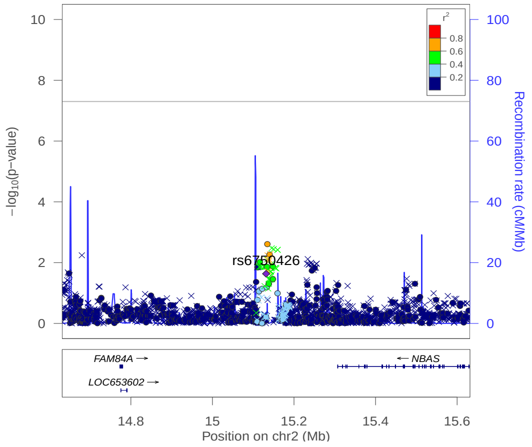

(e) South American

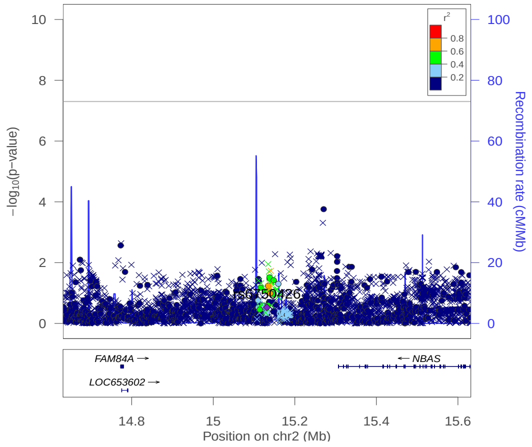

(f) Puerto Rican

Figure S11: Regional association plots across the Hispanic/Latino background subgroups for SNP rs6750426, detected in the Dominican subgroup.

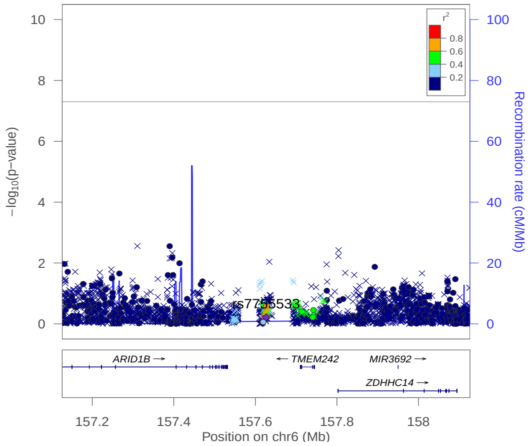

(a) Central American

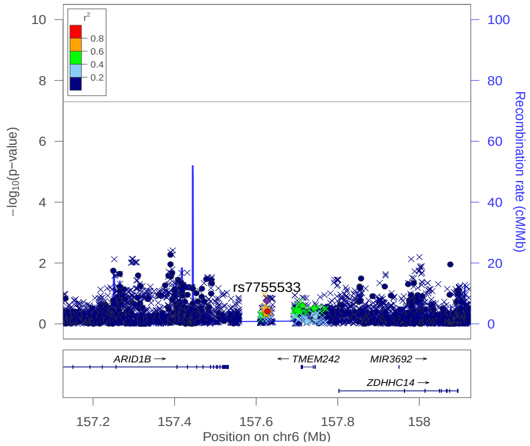

(b) Cuban

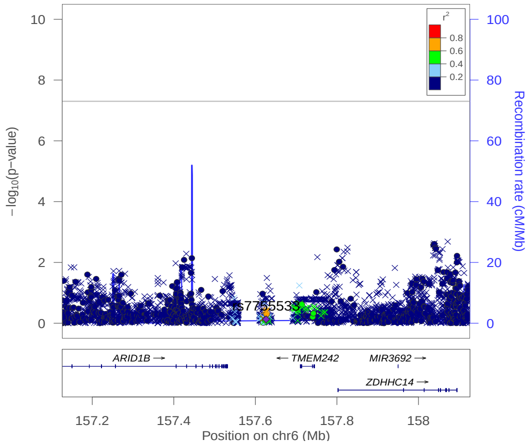

(c) Mexican

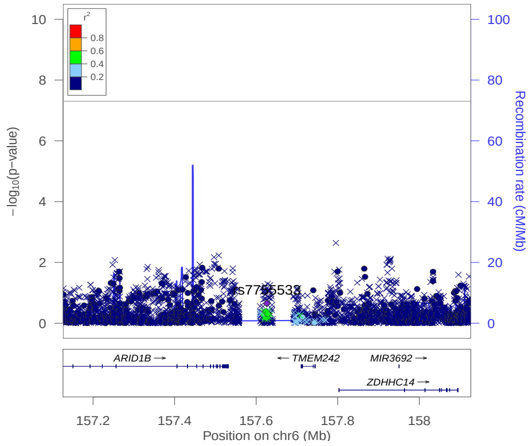

(d) Dominican

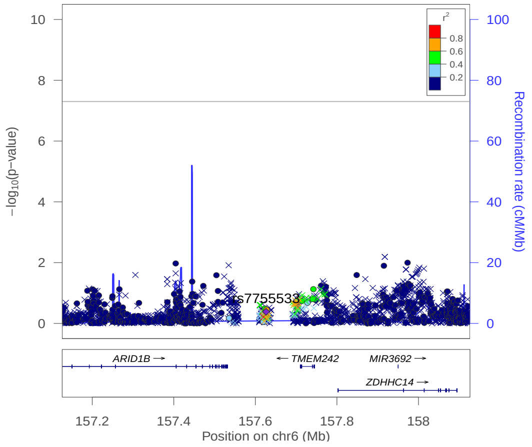

(e) South American

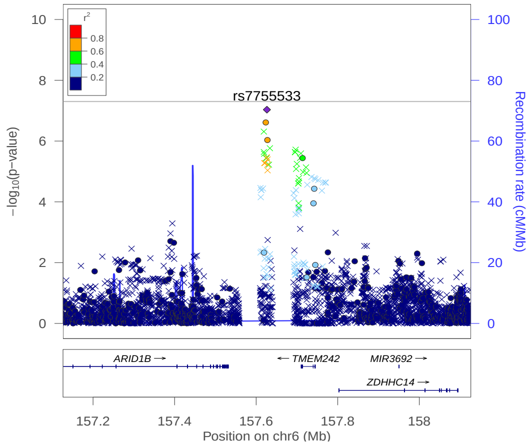

(f) Puerto Rican

Figure S12: Regional association plots across the Hispanic/Latino background subgroups for SNP rs7755533, detected in the Puerto-Rican subgroup.

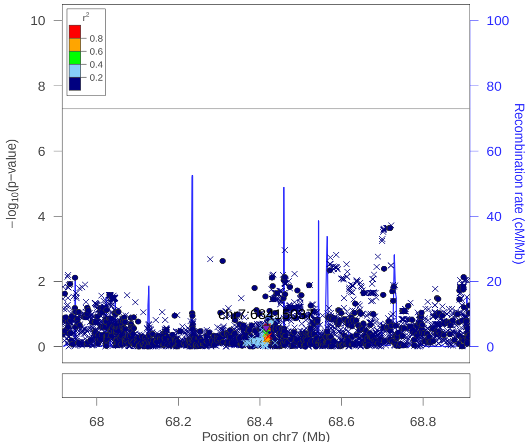

(a) Central American

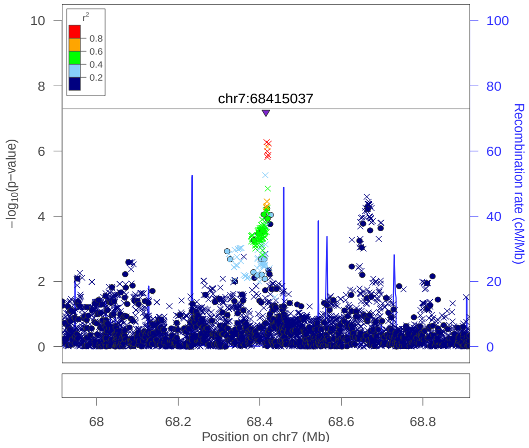

(b) Cuban

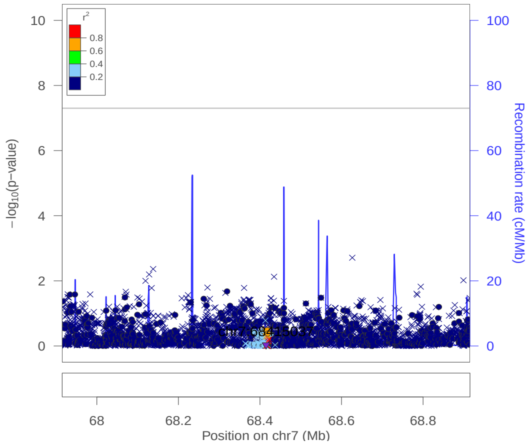

(c) Mexican

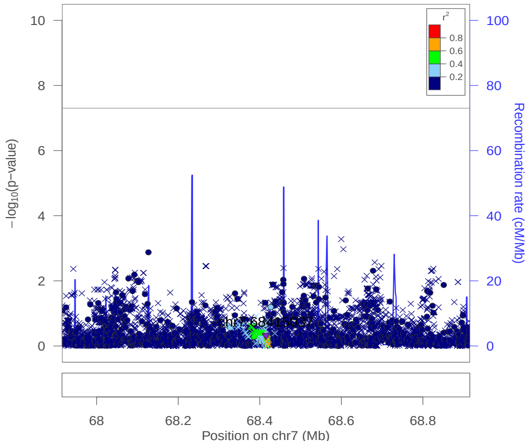

(d) Dominican

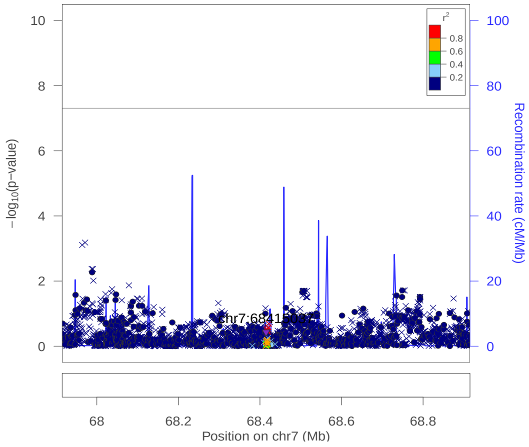

(e) South American

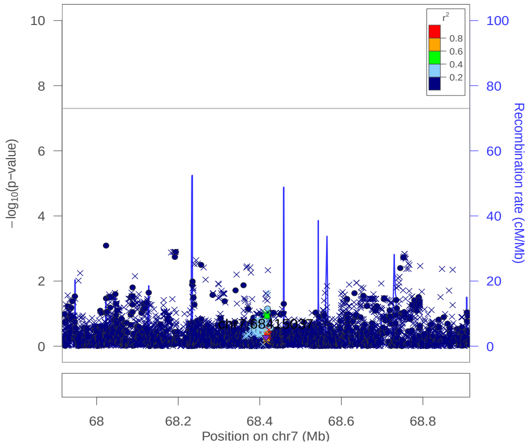

(f) Puerto Rican

Figure S13: Regional association plots across the Hispanic/Latino background subgroups for SNP rs113916643, detected in the Cuban subgroup.

2.6 Generalization of known SNP-trait associations

study	pop	rsID	Chr	position	A1	A2	Dscvr Beta	Dscvr p-value	SOL Beta	SOL p-value
Wassel et al. (2012)	AA	rs2243100	17	4779777	C	T	1.10E-02	5.00E-05	-7.80E-04	0.71
Wassel et al. (2012)	AA	rs2243093	17	4776675	T	C	1.20E-02	5.94E-05	-2.22E-04	0.91
Wassel et al. (2012)	AA	rs2660896	12	94947913	C	T	-1.00E-02	7.94E-05	-5.28E-04	0.75
Wassel et al. (2012)	AA	rs2242406	16	74131531	C	T	-3.40E-02	9.46E-05	1.21E-03	0.55
Wassel et al. (2012)	EA	rs2171209	6	159103550	C	T	-7.00E-03	6.02E-07	6.97E-04	0.68
Wassel et al. (2012)	EA	rs290481	10	114913815	C	T	-8.00E-03	7.01E-07	1.78E-03	0.38
Wassel et al. (2012)	EA	rs11061318	12	130135058	C	T	-1.60E-02	4.46E-06	-8.51E-03	0.10
Murabito et al. (2011)	EA	rs10757269	9	22062264	G	A	-4.90E-03	2.65E-09	-6.93E-04	0.67
Murabito et al. (2011)	EA	rs4659996	1	238912747	A	G	-4.50E-03	2.12E-06	-2.26E-03	0.16
Murabito et al. (2011)	EA	rs7003385	8	41705907	T	C	-4.30E-03	1.11E-06	3.66E-04	0.84
Murabito et al. (2011)	EA	rs819750	1	99469651	G	T	-4.10E-03	1.01E-03	-2.05E-03	0.51
Murabito et al. (2011)	EA	rs9485528	6	102221473	A	G	-4.10E-03	1.77E-04	3.52E-03	0.07
Murabito et al. (2011)	EA	rs722453	7	84037497	G	A	-5.20E-03	1.02E-06	-1.82E-03	0.27
Murabito et al. (2011)	EA	rs16824978	2	211380306	T	C	-3.90E-03	1.48E-04	-9.29E-04	0.59

Table S8: Generalization analysis of previously reported SNP associations with ABI. None of the associations generalized to Hispanics/Latinos. “pop” is the population studied in the discovery study, positions are given in build 37. All generalization r-values were 1, and are not shown.

Table S9: Generalization analysis of previously reported SNP associations with PAD. None of the associations generalized to Hispanics/Latinos. "pop" is the population studied in the discovery study, positions are given in build 37. Beta directions are 1 if the SNP is associated with increased odds or disease, and -1 otherwise. All generalization r-values were 1, and are not shown.

study	pop	rsID	Chr	pos	A1	A2	Dscvr Beta direction	Dscvr p-value	SOL Beta direction	SOL p-value
Wassel et al. (2012)	AA	rs4987756	18	59060091	A	G	1	3.78E-06	-1	0.83
Wassel et al. (2012)	AA	rs1256143	14	63981380	C	T	1	1.43E-05	-1	0.11
Wassel et al. (2012)	AA	rs13004470	2	242159756	C	T	1	4.69E-05	1	0.35
Wassel et al. (2012)	AA	rs9830448	3	154349978	C	A	1	4.79E-05	1	0.60
Wassel et al. (2012)	EA	rs11088283	21	34745649	A	G	-1	4.88E-05	1	0.38
Wassel et al. (2012)	EA	rs3745274	19	46204681	G	T	1	4.99E-05	1	0.99
Wassel et al. (2012)	EA	rs12428227	13	109700293	A	G	1	5.20E-05	-1	0.40
Wassel et al. (2012)	EA	rs17151901	8	10290865	C	T	1	6.53E-05	1	0.13
Koriyama et al. (2010)	Jap	rs1902341	3	31795570	G	A	1	4.70E-07	1	0.28
Koriyama et al. (2010)	Jap	rs6779621	3	31807513	G	T	1	2.70E-06	1	0.12
Koriyama et al. (2010)	Jap	rs2168422	3	31804842	A	C	1	2.10E-05	1	0.44
Koriyama et al. (2010)	Jap	rs2045298	3	31799254	T	C	1	2.70E-05	1	0.89
Koriyama et al. (2010)	Jap	rs2554503	8	3824825	C	G	1	5.70E-05	1	0.95
Koriyama et al. (2010)	Jap	rs1483466	8	94151120	T	C	1	1.90E-04	1	0.89
Koriyama et al. (2010)	Jap	rs431537	5	19478541	T	A	1	1.00E-02	-1	0.39
Koriyama et al. (2010)	Jap	rs235243	1	12319994	T	G	1	4.00E-02	-1	0.08
Koriyama et al. (2010)	Jap	rs3765337	1	12342599	C	A	1	5.00E-02	-1	0.06
Koriyama et al. (2010)	Jap	rs7659075	4	36225967	C	T	1	2.70E-01	1	0.65
Koriyama et al. (2010)	Jap	rs16946196	18	4191055	A	C	1	1.30E-01	1	0.57
Koriyama et al. (2010)	Jap	rs17647070	18	33102339	C	T	1	2.90E-04	-1	0.16
Koriyama et al. (2010)	Jap	rs17832415	8	12715797	T	C	1	4.00E-02	-1	0.08
Koriyama et al. (2010)	Jap	rs1807019	18	48378106	C	A	1	7.00E-02	-1	0.72
Koriyama et al. (2010)	Jap	rs1847040	5	81938623	T	G	1	2.00E-02	-1	0.38

Table S9: Generalization analysis of previously reported SNP associations with PAD. None of the associations generalized to Hispanics/Latinos. "pop" is the population studied in the discovery study, positions are given in build 37. Beta directions are 1 if the SNP is associated with increased odds or disease, and -1 otherwise. All generalization r-values were 1, and are not shown.

study	pop	rsID	Chr	pos	A1	A2	Dscvr Beta direction	Dscvr p-value	SOL Beta direction	SOL p-value
Koriyama et al. (2010)	Jap	rs1916998	5	142074132	A	C	1	2.20E-04	1	0.68
Koriyama et al. (2010)	Jap	rs2291016	8	93577910	T	G	1	8.00E-04	-1	0.08
Koriyama et al. (2010)	Jap	rs2359536	10	20899608	C	T	1	1.50E-06	1	0.47
Koriyama et al. (2010)	Jap	rs6481686	10	30798642	G	C	1	3.80E-04	1	0.08
Koriyama et al. (2010)	Jap	rs7217914	17	69831434	C	T	1	2.20E-04	1	0.48
Koriyama et al. (2010)	Jap	rs994950	3	35995617	A	G	1	9.80E-04	1	0.98
Kullo et al. (2014)	AA	rs653178	12	112007756	C		1	6.46E-07	-1	0.76
Matsukira et al. (2015)	Jap	rs9584669	13	98363482	C		-1	6.78E-14	-1	0.44
Matsukira et al. (2015)	Jap	rs6842241	4	148400819	A		-1	5.32E-09	1	0.28
Matsukira et al. (2015)	Jap	rs2074633	7	19035920	C		1	8.43E-08	1	0.96
Murabito et al. (2011)	EA	rs6584389	10	102459392	C	A	1	2.34E-06	-1	0.59
Murabito et al. (2011)	EA	rs9998941	4	162544312	A	G	1	2.34E-06	1	0.83
Murabito et al. (2011)	EA	rs11751656	6	42751046	G	A	1	2.46E-06	1	0.73
Murabito et al. (2011)	EA	rs4535726	8	68938371	T	C	1	3.79E-06	1	0.60
Murabito et al. (2011)	EA	rs2090205	17	73897869	A	C	1	5.01E-06	1	0.19
Murabito et al. (2011)	EA	rs11933540	4	25729099	C	T	1	9.86E-06	-1	0.31

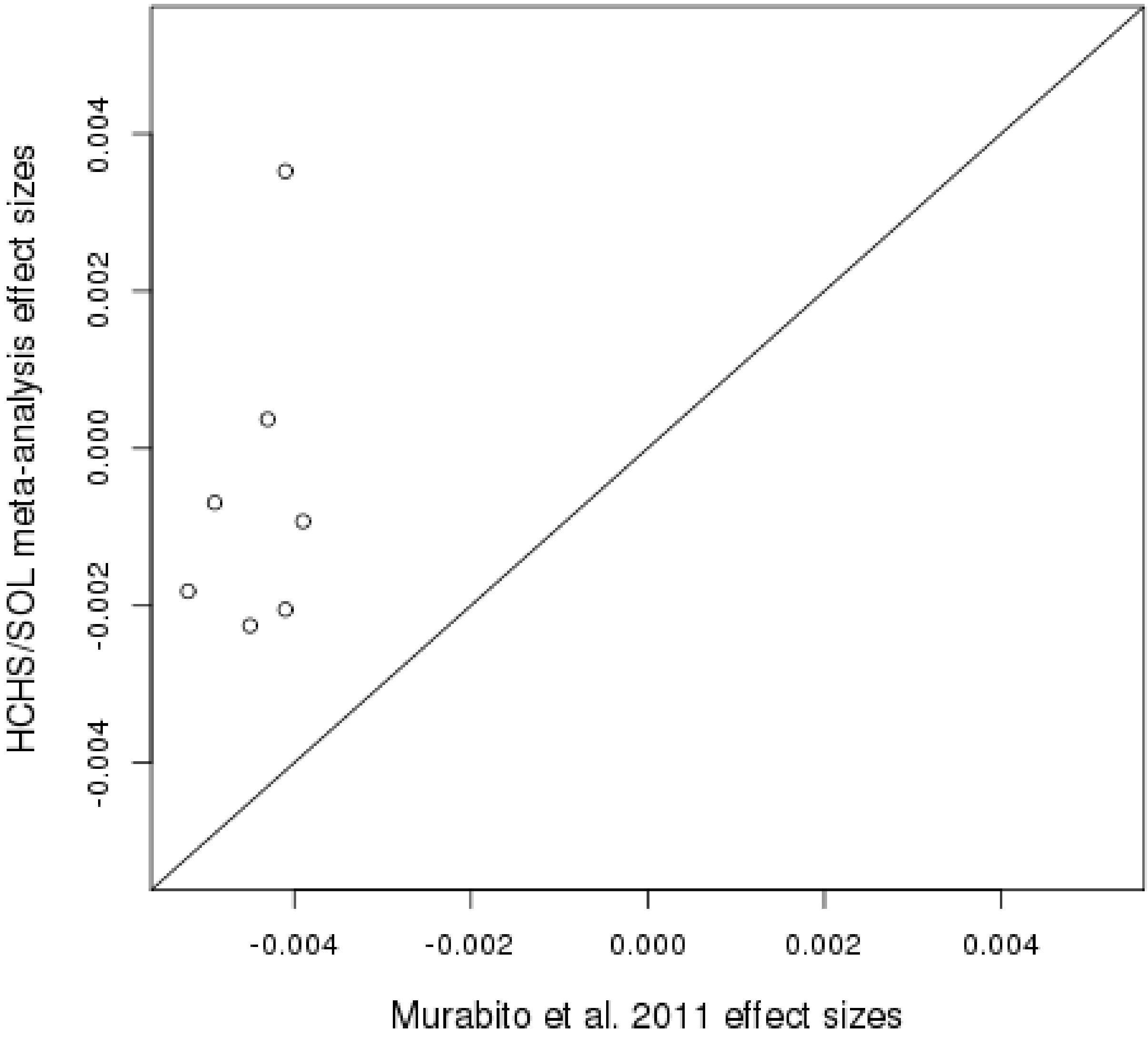

Figure S14: Comparison between the effect sizes of ABI of the SNPs reported in Murabito et al. 2011, and their estimated effect in the meta-analysis of all HCHS/SOL genetic analysis groups. Note of these SNP associations generalized to the HCHS/SOL Hispanics/Latinos, see Table S8.

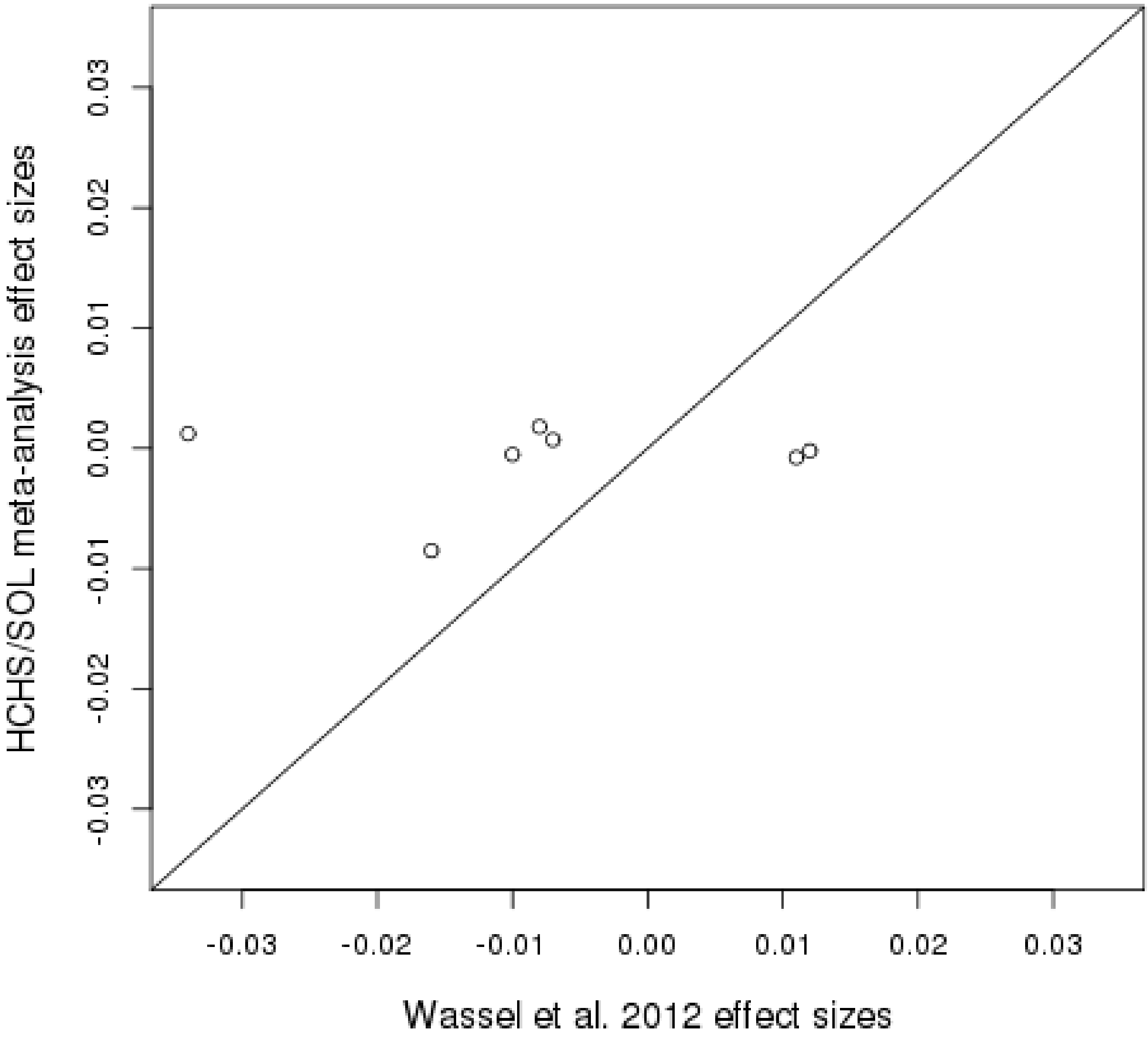

Figure S15: Comparison between the effect sizes of ABI of the SNPs reported in Wassel et al. 2012, and their estimated effect in the meta-analysis of all HCHS/SOL genetic analysis groups. Note of these SNP associations generalized to the HCHS/SOL Hispanics/Latinos, see Table S8.

2.7 Colocalization analysis of the region around rs4466200 with GTEx summary statistics

We performed colocalization analysis for the region around the SNP rs4466200 that was associated with ABI in the Puerto Rican group and was generally located in the *COMMD10* gene region. First, we identified all SNPs within a 1Mbp region centered at rs4466200 that had p-value¡0.001 in the Puerto Rican analysis. This resulted in 527 SNPs. Second, using Figure 2 (top) in the main manuscript we identified 6 potential genes, including *COMMD10*, that may overlap with the causal variant tagged by rs4466200. Specifically, we took: *COMMD10*, *QAPEP*, *ARL14EPL*, *LOC101927190*, *CTB-118N6.3*, and *SEMA6A*. Third, we identified six tissues available in GTEx (Consortium et al., 2015) version 7 and relevant to ABI: whole blood (Whole_Blood), aorta, coronary, and tibial arteries (Artery_Aorta, Artery_Coronary, Artery_Tibial), and the heart's arterial appendage and left ventricle (Heart_Atrial_Appendage, and Heart_Left_Ventricle).

Then, we performed colocalization analysis in the following manner: for each gene and tissue that of the lists defined above, if a significant eQTL existed in GTEx, and was also one of the 527 SNPs in the region around rs4466200 and with p-value¡0.001 in the Puerto Rican ABI analysis, we applied the function colo.abf from the "coloc" R package (Giambartolomei et al., 2014) with default parameters on the p-values from the ABI analysis and from the eQTL analysis, and with minor allele frequencies (MAFs) taken from the GTEx files. Figure S10 below compares the MAFs from all SNPs investigated in the colocalization analysis, between the GTEx data and the HCHS/SOL Puerto Ricans who participated in the ABI analysis, demonstrating that MAFs are similar and therefore colocalization analysis using our approach is appropriate. Analysis results are reported in Table S6 below and are further explained in the main manuscript results.

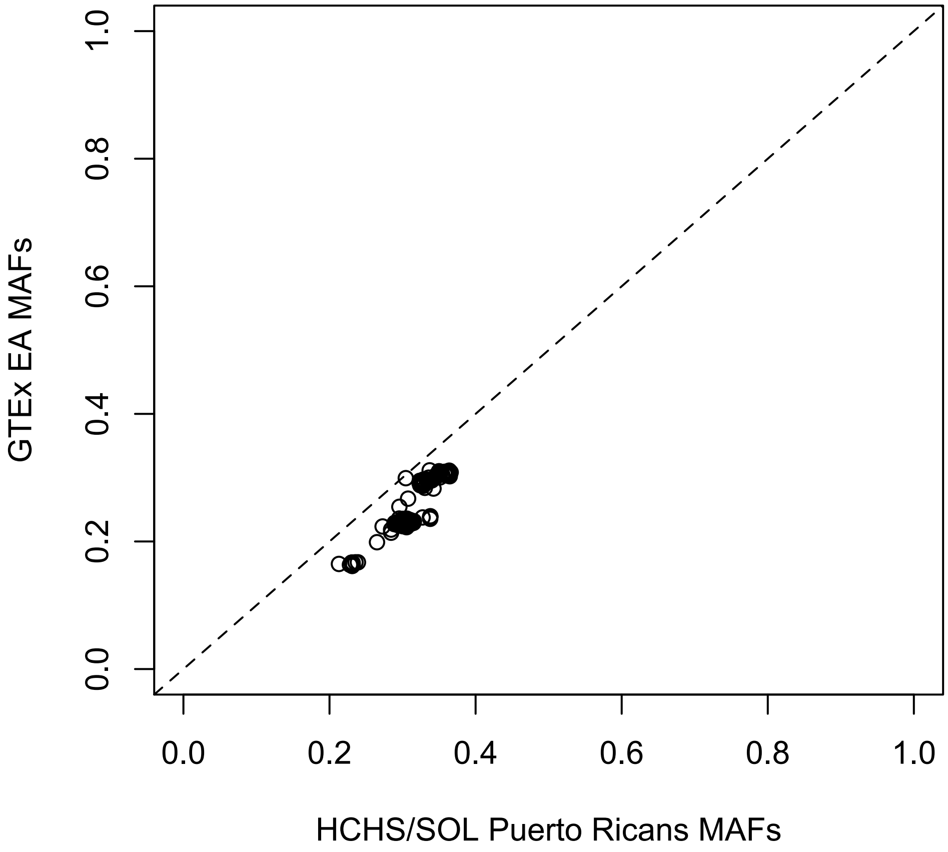

Figure S16: Comparisons of minor allele frequencies (MAFs) in GTEx and in HCHS/SOL Puerto Ricans for the common SNPs investigated in the colocalization analysis.

tissue	gene	P common SNP	most likely SNP	P likely SNP
Artery Aorta	*CTB-118N6.3*	0.00	rs10062588	0.31
Heart Atrial Appendage	*COMMD10*	0.10	rs4466200	0.08
Heart Left Ventricle	*COMMD10*	0.02	rs10062588	0.40
Heart Left Ventricle	*SEMA6A*	0.09	rs4466200	0.10

Table S10: Results from co-localization analysis of the region around rs4466200 using GTEx data. For each tissue and gene around the lead SNP that had significant eQTLs according to GTEx data (Consortium et al., 2015), and had overlapping significant eQLT SNPs with ABI SNPs with p-value< 0.001 in the Puerto Rican analysis, this table reports "P common SNP": the estimated probability using the coloc.abf function in the package "coloc" (Plagnol et al., 2008; Giambartolomei et al., 2014) that there is a shared causal SNP in the gene for the expression in the tissue and ABI, "most likely SNP": the SNP with the highest posterior probability to being causal for both expression and ABI, "P likely SNP" the posterior probability of being causal for both expression and ABI, for this SNP.

2.8 Pathway enrichment analysis

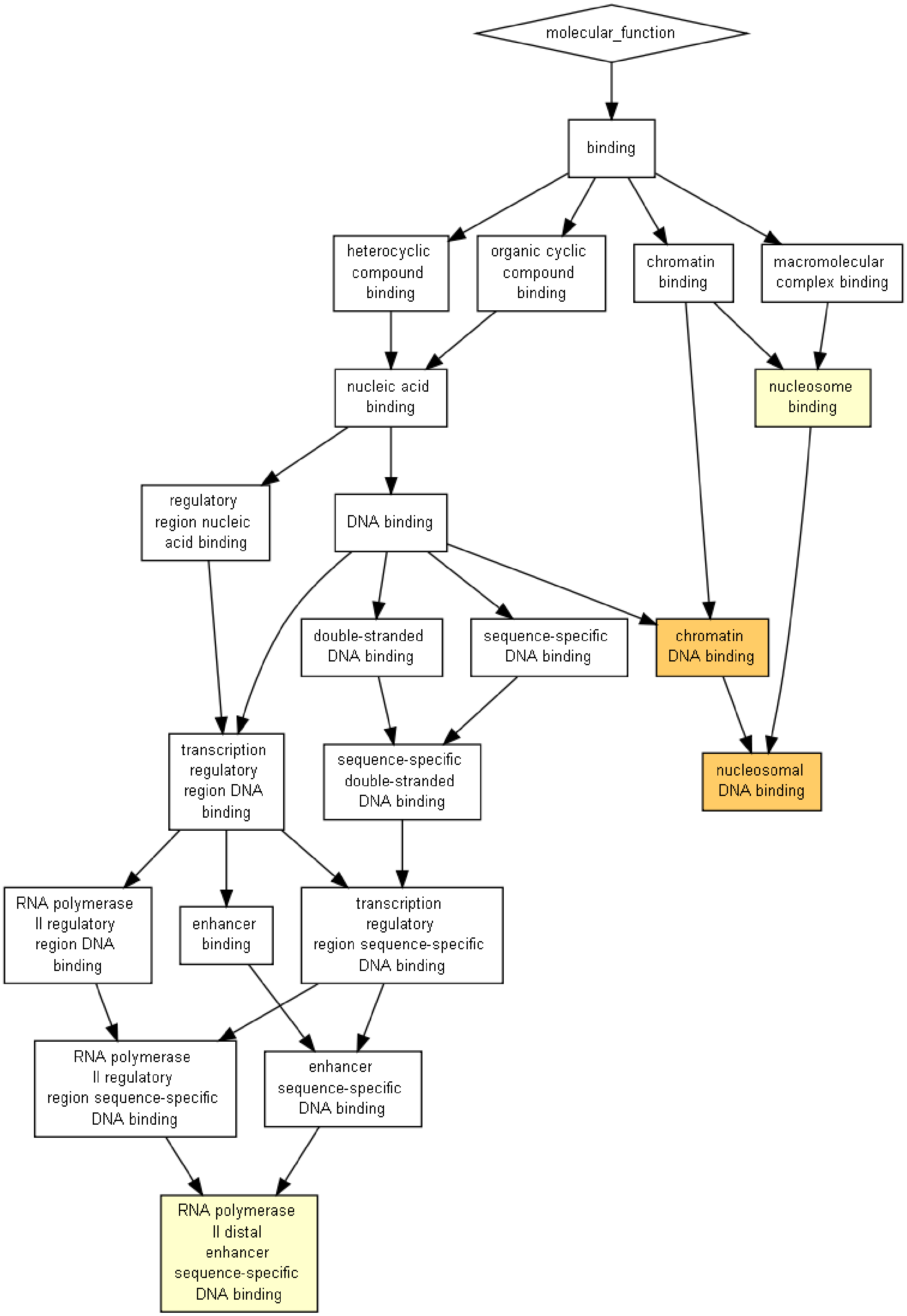

Figure S17: Visualization, produced by GOrilla (Eden et al., 2009b), of the pathway enrichment analysis for "function"-type pathways. The two pathways marked in orange were significant after controlling for False Discovery Rate (FDR).

3 Power calculations

We computed power for the GWAS of ABI: for the primary (pooled) analysis, for the Mainland and Caribbean groups, and all the Hispanic/Latino subgroups, using the analysis sample sizes. Figure S18 provides the computed powers for the genome-wide significance threshold (p-value¡5×10^{-8}), and Figure S19 provides the computed powers for higher threshold of 1×10^{-7}. The power were computed for SNP MAFs of 0.1, 0.3, and 0.5, and for a range of relatively high effect sizes (in GWAS terms), of 0.1,0.2, 0.2, and 0.4, 0.5 standard deviations of ABI.

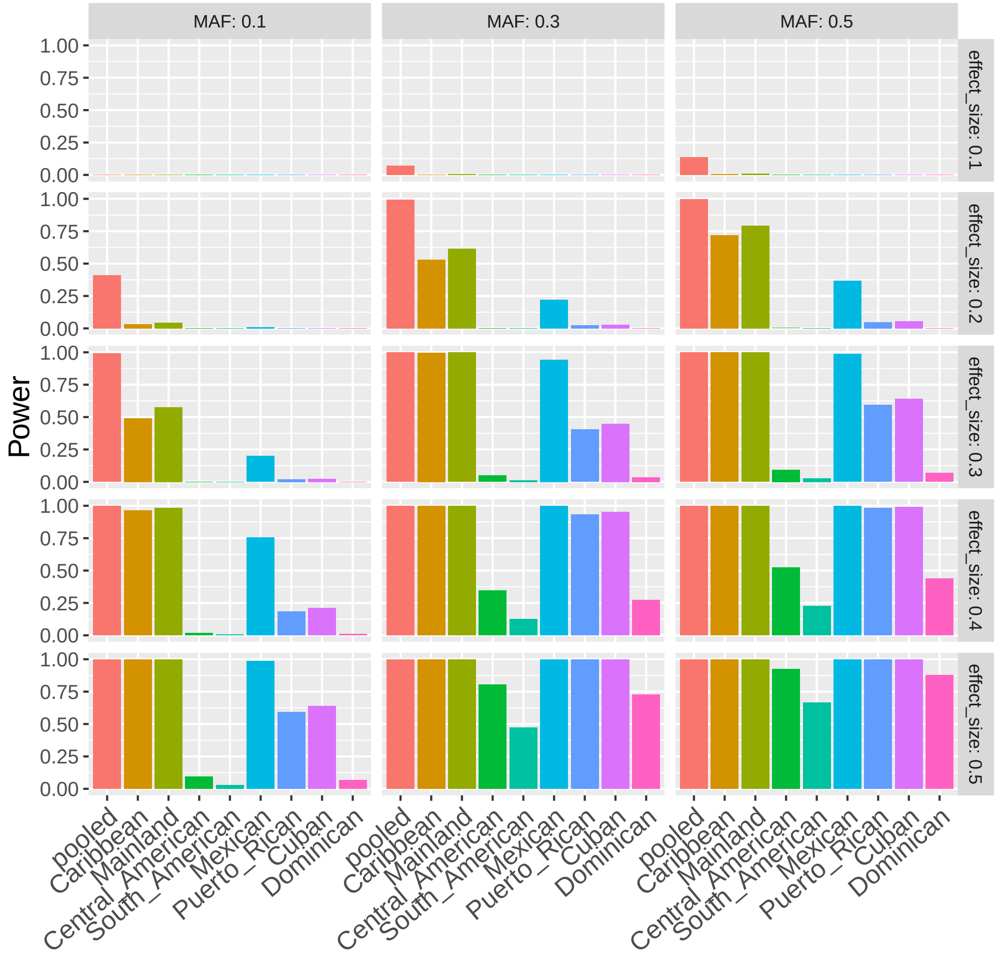

Figure S18: Power for detecting association based on p-value threshold of 5×10^{-8}, for a given analysis (based on the sample size of eligible individuals in the HCHS/SOL study), and a range of minor allele frequencies (MAFs) for common variants, and a range of effect sizes in terms of standard deviation of ABI.

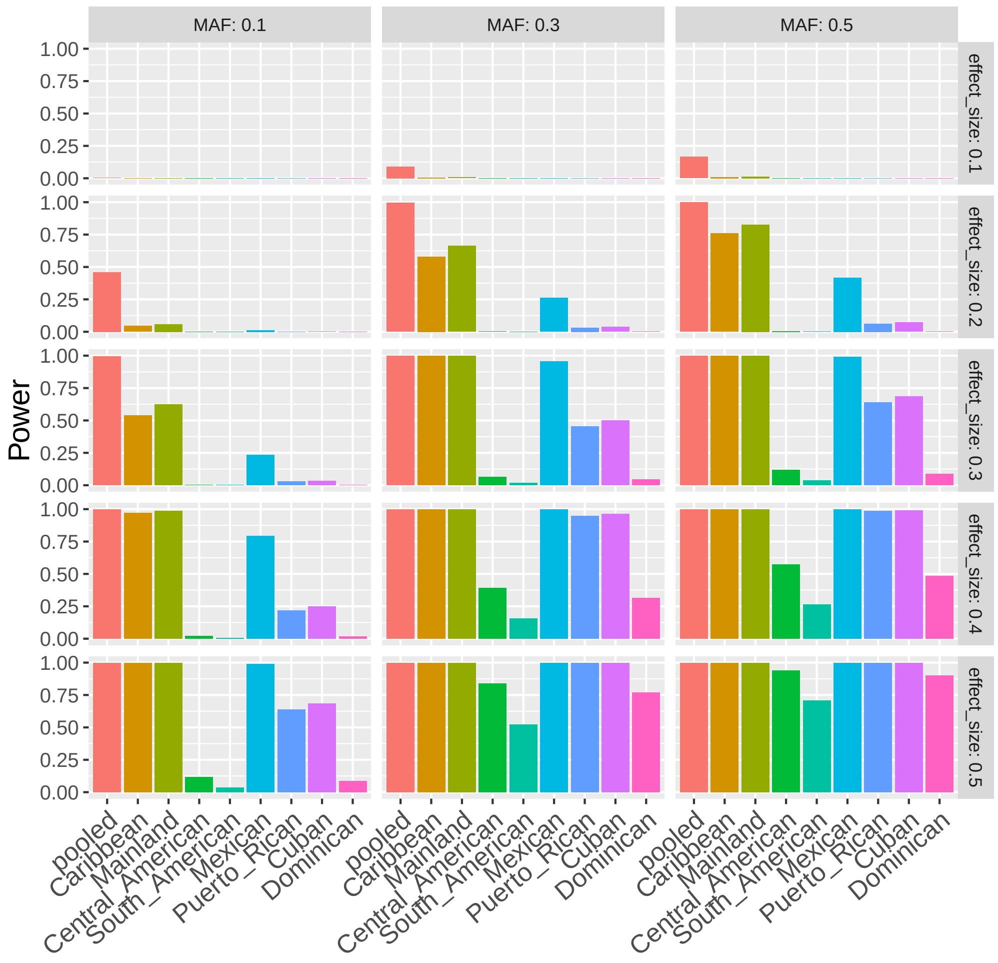

Figure S19: Power for detecting association based on p-value threshold of 5×10^{-8}, for a given analysis (based on the sample size of eligible individuals in the HCHS/SOL study), and a range of minor allele frequencies (MAFs) for common variants, and a range of effect sizes in terms of standard deviation of ABI.

4 Institutional Review Board that approved this study

4.1 IRBs that approved the HCHS/SOL

Non-Biomedical IRB at the University of North Carolina at Chapel Hill. Chapel Hill, NC.

Einstein IRB at the Albert Einstein College of Medicine of Yeshiva University. Bronx, NY.

IRB at Office for the Protection of Research Subjects (OPRS), University of Illinois at Chicago. Chicago, IL.

Human Subject Research Office, University of Miami. Miami, FL

Institutional Review Board of San Diego State University. San Diego, CA.

4.2 IRBs that approved MESA

Human Subjects Division at the University of Washington. Seattle, WA

Columbia University Institutional Review Board. New York, NY.

John Hopkins Medicine Institutional Review Board, Office of Human Subjects Research. Baltimore, MD.

University of Minnesota, Research Subjects? Protection Programs, IRB: Human Subjects Committee. Minneapolis, MN.

Institutional Review Board of Northwestern University - Office for the Protection of Research Subjects. Chicago, IL.

UCLA Institutional Review Board. Los Angeles, CA.

Institutional Review Board of Wake Forest School of Medicine, The Bowman Gray Campus. Winston-Salem, NC.

5 Reproducibility statement

All GWAS were performed and tracked using the Integrated Computing and Tracking (ICT) system of the HCHS/SOL genetic analysis center (Stilp et al., 2017). The unique GWAS analysis IDs are provided in Table S10.

Analysis	Analysis ID
ABI (pooled)	562063
ABI (stratified)	s163771
PAD	735273
Borderline PAD	866407

Table S11: Analysis IDs of the performed GWAS in the HCHS/SOL GAC tracking database.

References

Adams, D., Altucci, L., Antonarakis, S. E., Ballesteros, J., Beck, S., Bird, A., Bock, C., Boehm, B., Campo, E., Caricasole, A. et al. (2012). BLUEPRINT to decode the epigenetic signature written in blood. *Nature biotechnology*, **30** 224–226.

Carithers, L. J. and Moore, H. M. (2015). The genotype-tissue expression (GTEx) project.

Conomos, M., Laurie, C., Stilp, A., Gogarten, S., McHugh, C., Nelson, S., Sofer, T., Fernandez-Rhodes, L., Justice, A., Graff, M., Young, K., Seyerle, A., Avery, C., Taylor, K., Rotter, J., Talavera, G., Daviglus, M., Wassertheil-Smoller, S., Schneiderman, N., Heiss, G., Kaplan, R., Franceschini, N., Reiner, A., Shaffer, J., Barr, R., Kerr, K., Browning, S., Browning, B., Weir, B., Avilés-Santa, M., Papanicolaou, G., Lumley, T., Szpiro, A., North, K., Rice, K., Thornton, T. and Laurie, C. (2016). Genetic Diversity and Association Studies in US Hispanic/Latino Populations: Applications in the Hispanic Community Health Study/Study of Latinos. *The American Journal of Human Genetics*, **98** 165 – 184.

Consortium, E. P. et al. (2012). An integrated encyclopedia of DNA elements in the human genome. *Nature*, **489** 57.

Consortium, G. et al. (2015). The Genotype-Tissue Expression (GTEx) pilot analysis: Multitissue gene regulation in humans. *Science*, **348** 648–660.

Delaneau, O., Zagury, J.-F. and Marchini, J. (2013). Improved whole-chromosome phasing for disease and population genetic studies. *Nature methods*, **10** 5–6.

Dewey, M. (2019). *metap: meta-analysis of significance values*. R package version 1.1.

Eden, E., Lipson, D., Yogev, S. and Yakhini, Z. (2007). Discovering motifs in ranked lists of DNA sequences. *PLoS computational biology*, **3** e39.

Eden, E., Navon, R., Steinfeld, I., Lipson, D. and Yakhini, Z. (2009a). GOrilla: a tool for discovery and visualization of enriched GO terms in ranked gene lists. *BMC bioinformatics*, **10** 48.

Eden, E., Navon, R., Steinfeld, I., Lipson, D. and Yakhini, Z. (2009b). GOrilla: a tool for discovery and visualization of enriched GO terms in ranked gene lists. *BMC bioinformatics*, **10** 48.

Gao, X., Becker, L. C., Becker, D. M., Starmer, J. D. and Province, M. A. (2010). Avoiding the high Bonferroni penalty in genome-wide association studies. *Genetic epidemiology*, **34** 100–105.

Gao, X., Starmer, J. and Martin, E. R. (2008). A multiple testing correction method for genetic association studies using correlated single nucleotide polymorphisms. *Genetic epidemiology*, **32** 361–369.

Giambartolomei, C., Vukcevic, D., Schadt, E. E., Franke, L., Hingorani, A. D., Wallace, C. and Plagnol, V. (2014). Bayesian test for colocalisation between pairs of genetic association studies using summary statistics. *PLoS genetics*, **10** e1004383.

Howie, B. N., Donnelly, P. and Marchini, J. (2009). A flexible and accurate genotype imputation method for the next generation of genome-wide association studies. *PLoS Genet*, **5** e1000529.

Kent, W. J., Sugnet, C. W., Furey, T. S., Roskin, K. M., Pringle, T. H., Zahler, A. M. and Haussler, D. (2002). The human genome browser at UCSC. *Genome research*, **12** 996–1006.

Koriyama, H., Nakagami, H., Katsuya, T., Sugimoto, K., Yamashita, H., Takami, Y., Maeda, S., Kubo, M., Takahashi, A., Nakamura, Y. et al. (2010). Identification of evidence suggestive of an association with peripheral arterial disease at the osbpl10 locus by genome-wide investigation in the japanese population. *Journal of atherosclerosis and thrombosis*, **17** 1054–1062.

Kullo, I., Shameer, K., Jouni, H., Lesnick, T. G., Pathak, J., Chute, C. G. and de Andrade, M. (2014). The ATXN2-SH2B3 locus is associated with peripheral arterial disease: an electronic medical record-based genome-wide association study. *Frontiers in Genetics*, **5**. URL `http://www.frontiersin.org/applied_genetic_epidemiology/10.3389/fgene.2014.00166/abstract`.

Kundaje, A., Meuleman, W., Ernst, J., Bilenky, M., Yen, A., Kheradpour, P., Zhang, Z., Heravi-Moussavi, A., Liu, Y., Amin, V. et al. (2015). Integrative analysis of 111 reference human epigenomes. *Nature*, **518** 317.

Laurie, C. et al. (2010). Quality control and quality assurance in genotypic data for genome-wide association studies. *Genetic Epidemiology*, **34** 591–602.

Matsukura, M., Ozaki, K., Takahashi, A., Onouchi, Y., Morizono, T., Komai, H., Shigematsu, H., Kudo, T., Inoue, Y., Kimura, H. et al. (2015). Genome-Wide Association Study of Peripheral Arterial Disease in a Japanese Population. *PloS one*, **10** e0139262.

Murabito, J. M., White, C. C., Kavousi, M., Sun, Y. Y., Feitosa, M. F., Nambi, V., Lamina, C., Schillert, A., Coassin, S., Bis, J. C. et al. (2011). Association between chromosome 9p21 variants and the ankle-brachial index identified by a meta-analysis of 21 genome-wide association studies. *Circulation: Cardiovascular Genetics* CIRCGENETICS–111.

Plagnol, V., Smyth, D. J., Todd, J. A. and Clayton, D. G. (2008). Statistical independence of the colocalized association signals for type 1 diabetes and RPS26 gene expression on chromosome 12q13. *Biostatistics*, **10** 327–334.

Sofer, T. (2017). Confidence Intervals for Heritability via Haseman-Elston Regression. *Statistical Application in Genetics and Molecular Biology*, **16** 259–273.

Sofer, T., Heller, R., Bogomolov, M., Avery, C. L., Graff, M., North, K. E., Reiner, A., Thornton, T. A., Rice, K., Benjamini, Y., Laurie, C. C. and Kerr, K. F. (2017). A powerful statistical framework for generalization testing in GWAS, with application to the HCHS/SOL. *Genetic epidemiology*, **41** 251–258.

Sofer, T., Zheng, X., Gogarten, S. M., Laurie, C. A., Grinde, K., Shaffer, J. R., Shungin, D., O?Connell, J. R., Durazo-Arvizo, R. A., Raffield, L. et al. (2019). A fully adjusted two-stage procedure for rank-normalization in genetic association studies. *Genetic epidemiology*.

Stilp, A. M., Gogarten, S. M., Laurie, C. C. and Sofer, T. (2017). Integrated Computing And Tracking System For Centralized High-Throughput Genetic Analysis: A Case Study. *bioRxiv* 137596.

Ward, L. D. and Kellis, M. (2011). HaploReg: a resource for exploring chromatin states, conservation, and regulatory motif alterations within sets of genetically linked variants. *Nucleic acids research*, **40** D930–D934.

Wassel, C. L., Lamina, C., Nambi, V., Coassin, S., Mukamal, K. J., Ganesh, S. K., Jacobs, D. R., Franceschini, N., Papanicolaou, G. J., Gibson, Q. et al. (2012). Genetic determinants of the ankle-brachial index: a meta-analysis of a cardiovascular candidate gene 50k snp panel in the candidate gene association resource (care) consortium. *Atherosclerosis*, **222** 138–147.

Zhou, X. and Stephens, M. (2014). Efficient multivariate linear mixed model algorithms for genome-wide association studies. *Nature methods*, **11** 407.
